# Three-Year Clinical Outcomes Based on Pre-Percutaneous Coronary Intervention Coronary Blood Flow Grade and Symptom-to-Balloon Time in Patients with Non-ST-Segment Elevation Myocardial Infarction

**DOI:** 10.3390/jcm12113654

**Published:** 2023-05-24

**Authors:** Yong Hoon Kim, Ae-Young Her, Seung-Woon Rha, Cheol Ung Choi, Byoung Geol Choi, Soohyung Park, Dong Oh Kang, Jung Rae Cho, Ji Young Park, Sang-Ho Park, Myung Ho Jeong

**Affiliations:** 1 Division of Cardiology, Department of Internal Medicine, Kangwon National University College of Medicine, Kangwon National University School of Medicine, Chuncheon 24289, Republic of Korea; hermartha1@gmail.com; 2 Cardiovascular Center, Korea University Guro Hospital, Seoul 08308, Republic of Korea; wmagpie@korea.com (C.U.C.); shp503@naver.com (S.P.); gelly9@naver.com (D.O.K.); 3 Cardiovascular Research Institute, Korea University College of Medicine, Seoul 02841, Republic of Korea; trv940@naver.com; 4 Cardiology Division, Department of Internal Medicine, Kangnam Sacred Heart Hospital, Hallym University College of Medicine, Seoul 07441, Republic of Korea; jrjoe@naver.com; 5 Division of Cardiology, Department of Internal Medicine, Cardiovascular Center, Nowon Eulji Medical Center, Eulji University, Seoul 01830, Republic of Korea; cisamoe@gmail.com; 6 Cardiology Department, Soonchunhyang University Cheonan Hospital, Cheonan 31151, Republic of Korea; matsalong@schmc.ac.kr; 7 Department of Cardiology, Cardiovascular Center, Chonnam National University Hospital, Gwangju 61469, Republic of Korea; myungho@chollian.net

**Keywords:** non-ST-segment elevation myocardial infarction, percutaneous coronary intervention, reperfusion

## Abstract

We compared the 3-year clinical outcomes according to the degree of pre-percutaneous coronary intervention thrombolysis in myocardial infarction flow grade (pre-PCI TIMI) and symptom-to-balloon time (SBT) individuals who underwent successful stent implantation with a diagnosis of non-ST-segment elevation myocardial infarction (NSTEMI). A total of 4910 patients with NSTEMI were divided into two groups: pre-PCI TIMI 0/1 (SBT < 48 h: *n* = 1328, SBT ≥ 48 h: *n* = 558) and pre-PCI TIMI 2/3 (SBT < 48 h: *n* = 1965, SBT ≥ 48 h: *n* = 1059). The primary outcome was a 3-year all-cause death rate, and the secondary outcome was the composite endpoint of 3-year all-cause death, recurrent MI, or any repeat revascularization rate. After adjustment, in the pre-PCI TIMI 0/1 group, the 3-year all-cause death (*p* = 0.003), cardiac death (CD, *p* < 0.001), and secondary outcome (*p* = 0.030) values were significantly higher in the SBT ≥ 48 h group than in the SBT < 48 h group. However, patients with pre-PCI TIMI 2/3 had similar primary and secondary outcomes, regardless of the SBT group. Within the SBT < 48 h group, the pre-PCI TIMI 2/3 group exhibited significantly higher rates of 3-year all-cause death, CD, recurrent MI, and secondary outcome values than the pre-PCI TIMI 0/1 group. Patients in the SBT ≥ 48 h group with either pre-PCI TIMI 0/1 or TIMI 2/3 had similar primary and secondary outcomes. Our results suggest that shortening the SBT may confer a survival benefit in patients with NSTEMI and those in the pre-PCI TIMI 0/1 group compared to those in the pre-PCI TIMI 2/3 group.

## 1. Introduction

Promptly opening the infarct-related artery (IRA) in patients with ST-segment elevation myocardial infarction (STEMI) reduces the mortality rate [1]. In many clinical studies targeting patients with acute myocardial infarction (AMI), the thrombolysis in myocardial infarction (TIMI) flow grade is used to investigate the relationship between coronary flow grade and clinical outcomes before and after percutaneous coronary intervention (PCI) [2,3]. In patients with STEMI, patients who presented with pre-PCI TIMI flow grade 2/3 (pre-PCI TIMI 2/3) experienced significantly lower 1-year mortality rates than those who presented with pre-PCI TIMI flow grade 0/1 [2]. However, the studies investigating the relationship between pre-PCI TIMI and long-term clinical outcomes in non-STEMI (NSTEMI) groups are limited, and the findings are inconclusive [3,4,5]. According to Bailleul et al.’s study [3], the mortality was comparable between the pre-PCI TIMI 0/1 and pre-PCI TIMI 2/3 groups over a three-year follow-up period (adjusted hazard ratio [aHR]: 0.79; 95% confidence interval [CI]: 0.56–1.11; *p* = 0.17). The research conducted by De Luca et al. [4] demonstrated that a decrease in pre-PCI TIMI flow did not have an effect on the survival rate at one year for individuals diagnosed with acute coronary syndrome (ACS). Stone et al. [5] reported that the presence of pre-PCI TIMI 3 flow in patients with STEMI was independently associated with increased odds of survival compared to those without pre-PCI TIMI 3 flow (odds ratio 2.1; *p* = 0.04). STEMI is caused by acute total occlusion of the culprit artery leading to transmural ischemia, whereas NSTEMI is caused by temporary or incomplete coronary occlusion, resulting in non-transmural ischemia [6]. Therefore, the effect of pre-PCI TIMI on post-PCI outcomes may differ between patients with STEMI and those with NSTEMI, and further research is required to investigate this issue. In patients with AMI, the total ischemic time consists of symptom-to-door time (SDT) and door-to-balloon time (DBT) [7]. In patients with STEMI, some reports suggested that reducing total ischemic time is more important for reducing mortality and decreasing infarct size than reducing DBT [7,8]. Despite the recent guideline [9] advocating for an early invasive strategy for NSTEMI patients with at least one high-risk criterion, recent studies [10,11] have produced contradictory findings. Recently, Cha et al. [12] suggested that delayed hospitalization (SDT ≥ 24 h) was related to higher 3-year all-cause death (adjusted hazard ratio (aHR), 1.35; *p* < 0.001) compared with those without delayed hospitalization in patients with NSTEMI. In their study [12], DBT was not a significant determinant of the major clinical outcomes. The aim of this study was to examine the effects of pre-PCI TIMI grades (pre-PCI TIMI 0/1 or 2/3) and total ischemic time on the long-term prognosis of NSTEMI patients. We compared the 3-year outcomes based on the pre-PCI TIMI and symptom-to-balloon time ((SBT) < 48 h or ≥48 h).

## 2. Methods

### 2.1. Study Population

**Figure 1:**
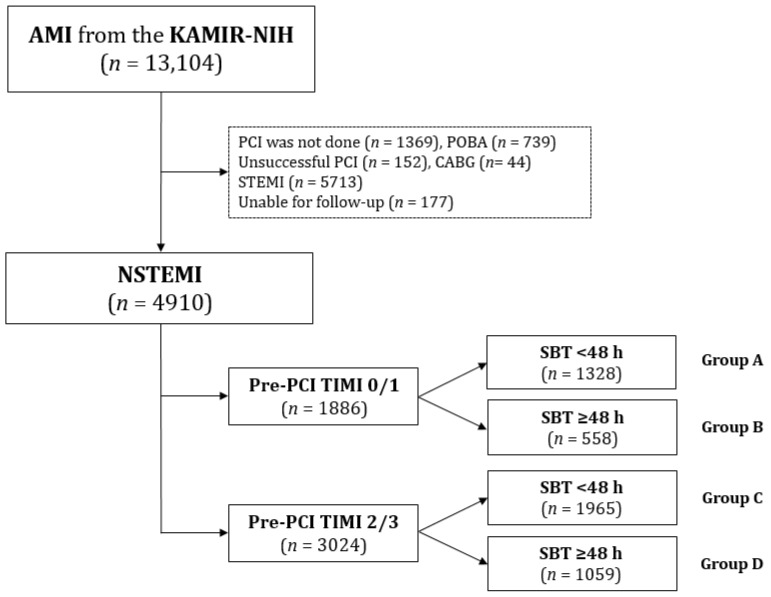
Flowchart. AMI, acute myocardial infarction; KAMIR-NIH, Korea Acute Myocardial Infarction Registry-National Institute of Health; PCI, percutaneous coronary intervention; POBA, plain old balloon angioplasty; CABG, coronary artery bypass graft; STEMI, ST-segment-elevation myocardial infarction; NSTEMI non-STEMI; Pre-PCI TIMI, pre-percutaneous coronary intervention thrombolysis in myocardial infarction flow grade; SBT, symptom-to-balloon time.

This cohort study was based on a multicenter prospective registry, the Korea Acute Myocardial Infarction Registry-National Institute of Health (KAMIR-NIH) [13]. From November 2011 to December 2015, 13,104 patients with AMI were registered in the KAMIR-NIH and were selected as the participants of the study. Twenty universities and teaching hospitals in the Republic of Korea participated in the KAMIR-NIH. Patients who were at least 18 years old at the time of enrollment were considered eligible for inclusion. Among the 13,104 patients, the analysis excluded individuals who did not undergo PCI (*n* = 1369, 10.4%), those who underwent plain old balloon angioplasty (*n* = 739, 5.6%), unsuccessful PCI (*n* = 152, 1.2%), coronary artery bypass graft (CABG, *n* = 44, 0.3%), those who had STEMI (*n* = 5713, 43.6%), and those who were lost to follow-up (*n* = 177, 1.4%) (Figure 1). Finally, 4910 patients with NSTEMI who underwent successful stent implantation were enrolled and classified into pre-PCI TIMI 0/1 (*n* = 1886, 38.4%) and pre-PCI TIMI 2/3 (*n* = 3024, 61.6%) groups. The two pre-PCI TIMI groups were subdivided into SBT < 48 h (groups A (*n* = 1328) and C (*n* = 1965)) and ≥48 h (groups B (*n* = 558) and D (*n* = 1059)) subgroups (Figure 1). The Ethics Committee at each participating center granted approval for this non-randomized study as well as the Chonnam National University Hospital Institutional Review Board Ethics Committee (CNUH-2011-172) in accordance with the ethical guidelines of the 2004 Declaration of Helsinki. Written informed consent was obtained from all 4910 patients before enrollment was possible in this study. These patients successfully completed a 3-year clinical follow-up through in-person visits, telephone tracking, and review of medical records. An online system was utilized to register data from all the participating PCI centers. The event adjudication procedures were documented and discussed in a prior publication, and an independent event-adjudicating committee within the KAMIR-NIH monitored and evaluated the occurrence of all events [13].

### 2.2. Percutaneous Coronary Intervention and Medical Treatment

The protocols for coronary angiography (CAG) and PCI were based on well-established guidelines [14]. Prior to PCI, patients received loading doses of aspirin (200–300 mg) in combination with clopidogrel (300–600 mg), ticagrelor (180 mg), or prasugrel (60 mg). All patients were recommended to take aspirin 100 mg daily and, as dual antiplatelet therapy, either clopidogrel 75 mg or ticagrelor 90 mg twice daily or prasugrel 5–10 mg for at least 1 year after PCI. The operators had the freedom to select the site of access, the strategy for revascularization, and the type of stent to be used.

### 2.3. Study Definitions and Clinical Outcomes

The diagnostic criteria for NSTEMI were based on the guidelines presented in the fourth universal definition of MI [15]. Successful PCI was defined as less than 30% residual stenosis in the IRA with a TIMI flow grade 3. The Global Registry of Acute Coronary Events (GRACE) risk score [16] for all patients was calculated. The time of onset of the last sustained chest pain was defined as the time of symptom onset in each patient [17]. There is limited evidence regarding the clinical outcomes of patients who experienced symptoms for more than 24 h before seeking medical attention, and Cha et al. [12] defined delayed hospitalization as when patients seek medical attention at the hospital ≥ 24 h after symptom onset (SDT ≥ 24 h). Guidelines [9,18] define “CAG performed within 24 h of hospital admission with intent to perform revascularization if appropriate based on coronary anatomy” as an “early invasive” approach. Therefore, we set a cut-off value of 48 h to divide the groups based on the SBT. The definition of typical chest pain utilized in this study encompassed substernal discomfort of a particular nature and duration, provoked by physical exertion or emotional stress, and improved by rest or the use of nitroglycerin [9]. Atypical chest pain was defined as chest pain that does not have the typical features of angina. The primary outcome of the present study was the rate of all-cause death during a 3-year follow-up period. The secondary outcome was the composite endpoint of all-cause death, recurrent MI, or any repeat revascularization during the same 3-year period. All deaths were classified as cardiac death (CD) unless an undisputed noncardiac cause was identified [19]. In this study, repeat revascularization was defined as target lesion revascularization, target vessel revascularization (TVR), or non-TVR. Recurrent MI, target lesion revascularization, TVR, and non-TVR were defined using the criteria established in previous studies [20].

### 2.4. Statistical Analyses

In order to conduct the statistical analyses, we utilized IBM’s Statistical Package for the Social Sciences (SPSS) software version 20, which is located in Armonk, NY, USA. Continuous variables were compared between groups using unpaired t-tests, and results were reported as either mean ± standard deviation or median (interquartile range). Categorical variables were analyzed using the chi-square test or Fisher’s exact test, and data were presented as counts and percentages. The SBT < 48 h and SBT ≥ 48 h groups were compared through univariate analyses of all variables, with a significance threshold of *p* < 0.05. Moreover, to ensure noncollinearity for all significant variables, multicollinearity tests [21] were performed (Table S1). The variance inflation factor (VIF) values were calculated to evaluate the level of multicollinearity among the variables. VIF values greater than 5 indicate a high degree of multicollinearity [22]. We considered the presence of multicollinearity when the tolerance value was under 0.1 [23] or when the condition index exceeded 10 [22]. The variables included in the multivariable analysis after undergoing statistical verification steps were selected as follows: male sex, age, left ventricular ejection fraction (LVEF), body mass index, cardiogenic shock, cardiopulmonary resuscitation (CPR) upon admission, SDT, DBT, atypical chest pain, dyspnea, Q-wave, and T-wave inversion on the electrocardiogram; Killip class II/III; emergency medical services, PCI center; diabetes mellitus; current smoker; levels of peak creatine kinase myocardial band (CK-MB) and troponin-I; and total cholesterol, low-density lipoprotein cholesterol, GRACE risk score, and clopidogrel (Table S1). To control for potential confounding variables, we performed a propensity score (PS)-adjusted analysis using a logistic regression model. All variables in Table 1 were included in the PS-matched analysis. The c-statistic for the PS-matched analysis in this study was 0.702. The matching of patients in the SBT ≥ 48 h group to those in the SBT < 48 h group was performed in a 1:1 fashion using the nearest available pair-matching method, and the measurement was performed using a caliper width of 0.01. Table S2 shows baseline characteristics between the SBT < 48 h and SBT ≥ 48 h groups before and after PS-matched analysis. Clinical outcomes were estimated using the Kaplan–Meier curve analysis, and group variances were compared using the log-rank test. Statistical significance was set at *p* < 0.05. The degree of multicollinearity was assessed for all-cause death between the pre-PCI TIMI 0/1 and pre-PCI TIMI 2/3 groups using a collinearity test, which is presented in Table S3.

## 3. Results

### 3.1. Baseline Characteristics

The baseline characteristics are shown in Tables S1, S2 and S4. Among patients in both the pre-PCI TIMI 0/1 and pre-PCI TIMI 2/3 groups, the number of patients who used emergency medical services, those who were current smokers, and the mean levels of peak CK-MB and troponin-I were higher in the SBT < 48 h group than in the SBT ≥ 48 h group. In contrast, the SBT ≥ 48 h group had a higher number of patients with atypical chest pain, a higher proportion of patients in Killip classes II and III, a higher mean age, a higher mean value of GRACE risk score, and a higher mean value of the deployed stent length compared to the SBT < 48 h group (Table 1). After the PS-matched analysis, 3058 matched pairs were identified (Table S2). In Table S4, among patients in both the SBT < 48 h and SBT ≥ 48 h groups, the pre-PCI TIMI 0/1 group had a significantly higher proportion of patients with Killip classes II/III, right coronary artery (RCA) as the IRA and treated vessel, second-generation drug-eluting stent (DES), use of glycoprotein IIb/IIIa inhibitors, peak CK-MB, troponin-I, total cholesterol, total stent length, and number of deployed stents compared to the pre-PCI TIMI 2/3 group. On the other hand, the pre-PCI TIMI 2/3 group had a higher proportion of patients with hypertension, left anterior descending artery (LAD), and left main coronary artery as the IRA and treated vessels, patients who underwent the transradial approach, mean LVEF, systolic blood pressure (SBP), and diameter of the deployed stent compared to the pre-PCI TIMI 0/1 group.

### 3.2. Clinical Outcomes

**Figure 2:**
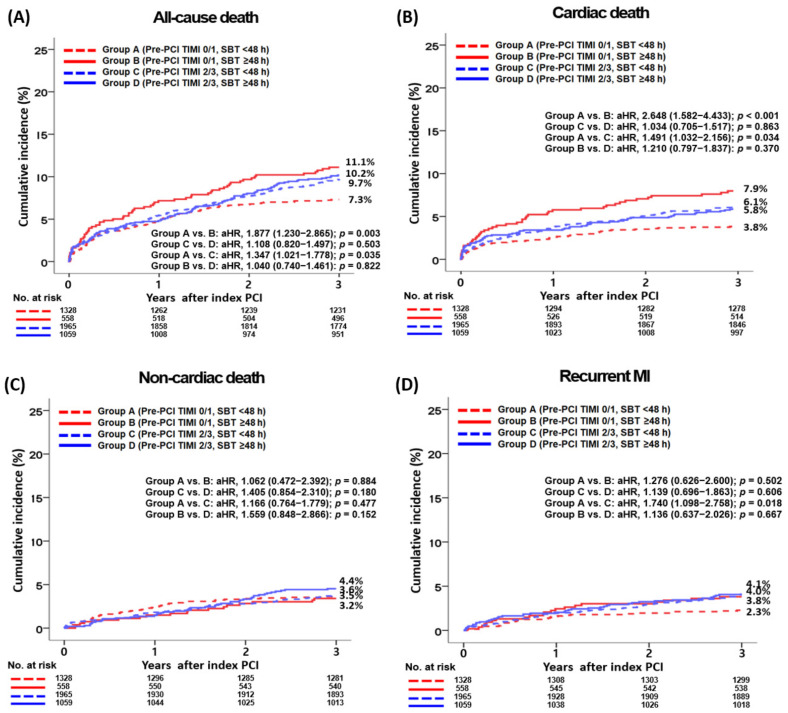

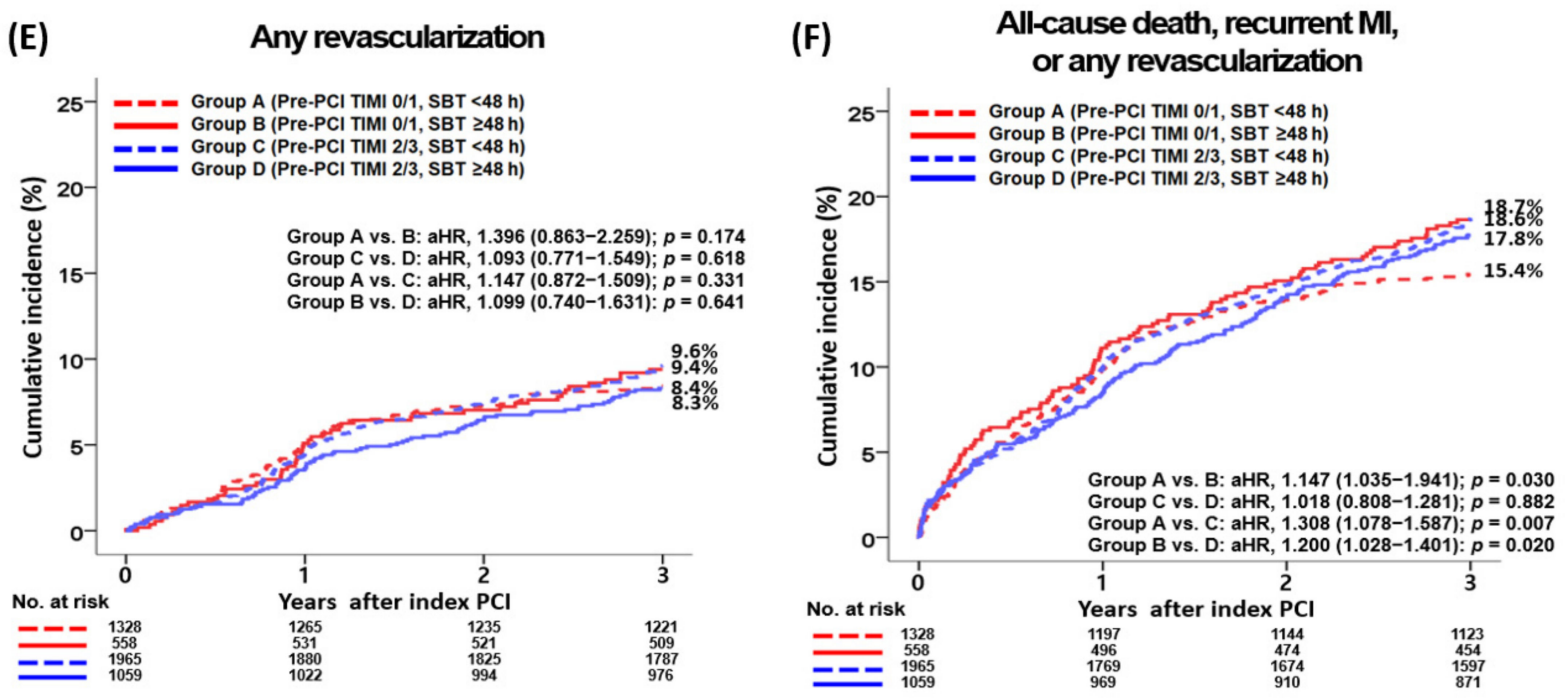
Kaplan–Meier analysis for all-cause death (**A**), cardiac death (**B**), non-cardiac death (**C**), recurrent MI (**D**), any repeat revascularization (**E**), and all-cause death, recurrent MI, or any repeat revascularization (**F**) during a 3-year follow-up period.

**Figure 3:**
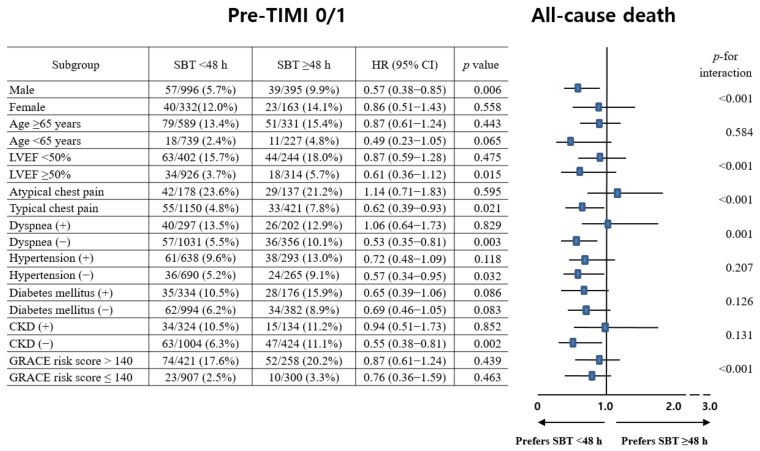

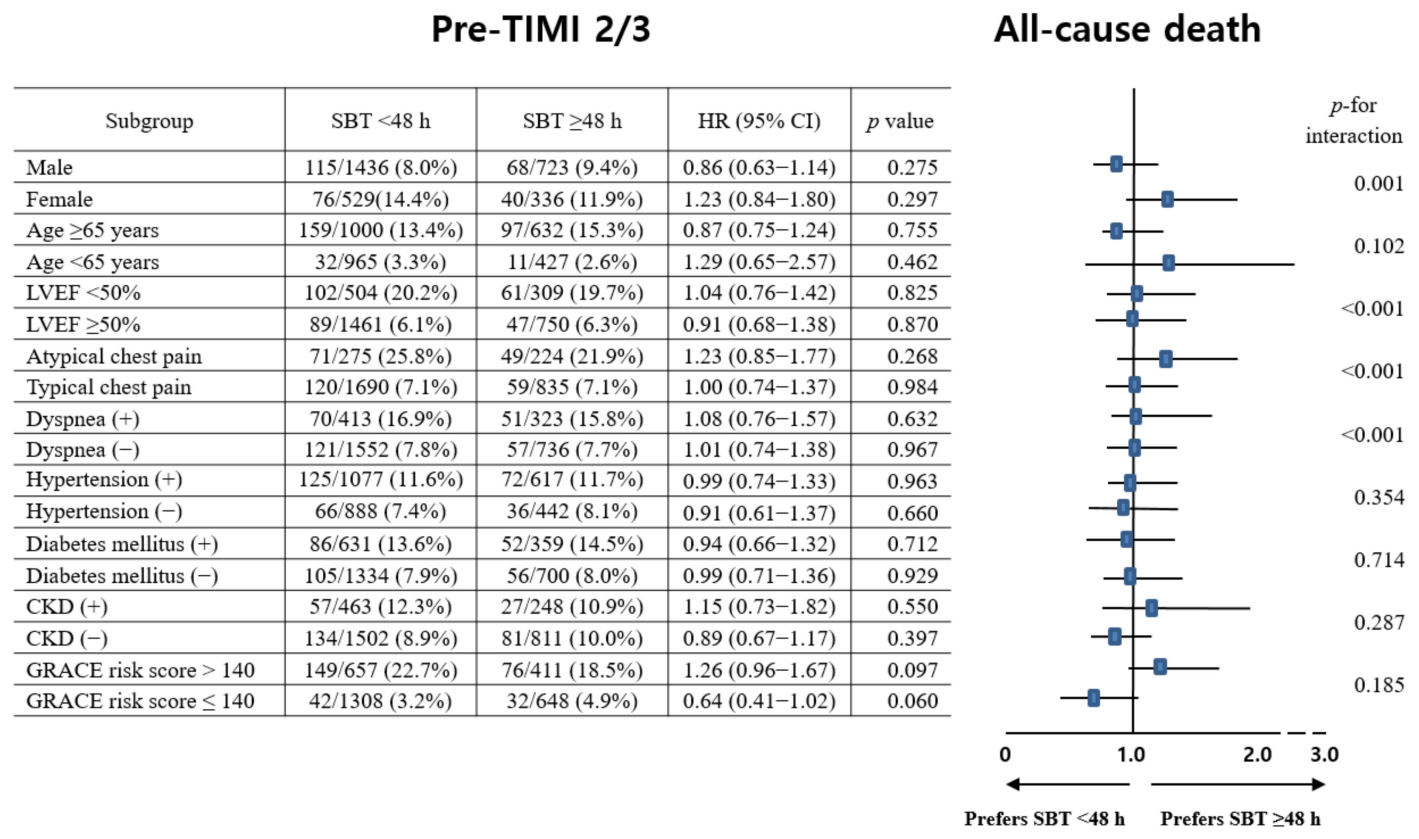
Subgroup analysis for all-cause death in the pre-PCI TIMI 0/1 and pre-PCI TIMI 2/3 groups. Pre-PCI TIMI, pre-percutaneous coronary intervention thrombolysis in myocardial infarction flow grade; HR, hazard ratio; CI, confidence interval; LVEF, left ventricular ejection fraction; CKD, chronic kidney disease; GRACE Global Registry of Acute Coronary Events.

The main results of the 3-year study are shown in Table 2 and Table 3, along with Figure 2. In the pre-PCI TIMI 0/1 group, all-cause death occurred in 7.3% of the patients at 3 years in the SBT < 48 h group and in 11.1% of patients at 3 years in the SBT ≥ 48 h group (aHR, 1.877; 95% CI, 1.230–2.865; *p* = 0.003). Moreover, CD (aHR, 2.648; 95% CI, 1.582–4.433; *p* < 0.001) and secondary outcome (composite endpoint of all-cause death, recurrent MI, or any repeat revascularization, aHR, 1.147; 95% CI, 1.035–1.941; *p* = 0.030) rates were significantly higher in the SBT ≥ 48 h group than in the SBT < 48 h group (Table 2). However, the CD (aHR, 1.062; *p* = 0.884), recurrent MI (aHR, 1.276; *p* = 0.502), and any repeat revascularization (aHR, 1.396; *p* = 0.174) rates were similar between the SBT < 48 h and SBT ≥ 48 h groups. In the pre-PCI TIMI 2/3 group, the rate of all-cause death was 9.7% in the SBT < 48 h group and 10.2% in the SBT ≥ 48 h group at 3 years, with an aHR of 1.108 (95% confidence interval [CI], 0.820–1.497; *p* = 0.503), indicating no significant difference between the two groups in terms of the primary outcome. The secondary outcome rate was also not significantly different between the SBT < 48 h and SBT ≥ 48 h groups. However, in the total study population, the SBT ≥ 48 h group had a significantly higher all-cause death rate (aHR, 1.278; *p* = 0.047) than the SBT < 48 h group (Table 2). The results remained the same even after PS-adjusted analyses were conducted (Table 2). In Table 3, after conducting multivariable-adjusted analyses, it was found that in the SBT < 48 h group, the pre-PCI TIMI 2/3 group had significantly higher rates of all-cause death (aHR, 1.347; *p* = 0.035), CD (aHR, 1.491; *p* = 0.034), recurrent MI (aHR, 1.740; *p* = 0.018), and secondary outcomes (aHR, 1.308; *p* = 0.007) compared to the pre-PCI TIMI 0/1 group. However, in the SBT ≥ 48 h group, there were no significant differences in the rates of primary and secondary outcomes between the pre-PCI TIMI 0/1 and pre-PCI TIMI 2/3 groups. Although there were no significant differences in the 3-year mortality rates between the pre-PCI TIMI 0/1 and pre-PCI TIMI 2/3 groups in the total study population, the pre-PCI TIMI 2/3 group exhibited significantly higher rates of recurrent MI (aHR, 1.486; *p* = 0.030) and secondary outcomes (aHR, 1.200; *p* = 0.020) compared to the pre-PCI TIMI 0/1 group after multivariable-adjusted analysis. Table 4 presents the factors that independently predict all-cause death. In both the pre-PCI TIMI 0/1 and pre-PCI TIMI 2/3 groups, advanced age (≥65 years old, *p* = 0.002 and *p* < 0.001, respectively), reduced left ventricular ejection fraction (<50%, *p* = 0.010 and *p* = 0.002, respectively), cardiopulmonary resuscitation upon admission (*p* < 0.001 and *p* < 0.001, respectively), atypical chest pain (*p* < 0.001 and *p* = 0.003, respectively), and high GRACE risk scores (>140, *p* < 0.001 and *p* < 0.001, respectively) were identified as significant independent predictors of all-cause death. Furthermore, in the pre-PCI TIMI 0/1 group, a SDT < 24 h (*p* = 0.033) and the left circumflex coronary artery (LCx) as the IRA (*p* = 0.020) were significant independent predictors of all-cause death. Figure 3 presents the results of the subgroup analyses of all-cause death in the pre-PCI TIMI 0/1 and pre-PCI TIMI 2/3 groups. In the pre-PCI TIMI 0/1 group, patients without hypertension (*p* = 0.032) or chronic kidney disease (*p* = 0.002) exhibited a higher all-cause death rate in the SBT ≥ 48 h group than in the SBT < 48 h group. However, in the pre-PCI TIMI 2/3 group, except for those with a significant *p*-for-interaction, comparable all-cause death rates were observed between the SBT < 48 h and SBT ≥ 48 h groups.

## 4. Discussion

From this prospective observational cohort study, we obtained the following results: First, in the pre-PCI TIMI 0/1 group, the SBT ≥ 48 h group was associated with a significantly higher risk of all-cause death, cardiac death, and secondary outcomes at 3 years compared to a SBT < 48 h group. However, in the pre-PCI TIMI 2/3 group, there was no significant difference in primary and secondary outcomes between the two groups. Second, in the SBT < 48 h group, the pre-PCI TIMI 2/3 group had a significantly higher risk of all-cause death, cardiac death, recurrent MI, and secondary outcomes at three years compared to the pre-PCI TIMI 0/1 group. However, in the SBT ≥ 48 h group, there was no significant difference in primary and secondary outcomes between the pre-PCI TIMI 0/1 and pre-PCI TIMI 2/3 groups. Third, the study findings revealed that advanced age, reduced LVEF, CPR on admission, atypical chest pain, and high GRACE risk scores were identified as significant independent predictors of all-cause death in both the pre-PCI TIMI 0/1 and pre-PCI TIMI 2/3 groups.

STEMI and NSTEMI differ in pathophysiology, treatment strategies, and outcomes [6,24]. The relationship between the timing of revascularization based on angiographic characteristics and clinical outcomes in patients with NSTEMI is not well understood [3,4,25]. A previous randomized controlled study [4] indicated that decreased baseline TIMI flow in moderate- and high-risk patients with ACS who underwent PCI did not affect their 1-year survival. According to a French registry [3], the incidence of pre-PCI TIMI 2/3 was higher in patients with NSTEMI, while it was an independent prognostic factor for both short- and long-term survival in patients with STEMI; however, it did not have a significant association with early or long-term survival in patients with NSTEMI. The pre-PCI TIMI 2/3 group constituted the majority of the study population, accounting for 61.6% (3024/4910) of all patients enrolled in this study (Figure 1), and the study results indicated that there were no statistically significant differences in the rates of all-cause death and cardiac death between the pre-PCI TIMI 0/1 and pre-PCI TIMI 2/3 groups (*p* = 0.102 and *p* = 0.148, respectively; Table 3).

In the De Luca study [4], CAG was performed 72 h after randomization, whereas in the French registry study [3], PCI was classified according to the DBT. However, notably, in our study, the 3-year outcome was selectively derived not only based on pre-PCI TIMI but also according to SBT. Furthermore, in our study, unlike the pre-PCI TIMI 2/3 group, the pre-PCI TIMI 0/1 group showed a different pattern of results. Specifically, the SBT < 48 h group had a lower 3-year mortality rate compared to the SBT ≥ 48 h group. Although further study is required to confirm these results, there are several possible explanations. Delayed or missed acute reperfusion resulting from failure or delay in recognizing acute LCx occlusion in patients with NSTE-ACS has been associated with poor outcomes [26]. The sensitivity of electrocardiography for detecting a total obstruction in the inferolateral distribution was reduced. Hence, in this case, patients with NSTEMI may be a subset of those with STEMI who were not effectively identified using 12-lead electrocardiogram screening [27]. Patients with NSTEMI and total obstruction of the coronary artery without STE have shown poorer outcomes than those with STEMI who present with the same obstruction but have characteristic STE, requiring timely revascularization [27,28]. Similar to previous results [26,27,28], in our study, the frequency of LCx as the IRA in the pre-PCI TIMI 0/1 group was higher than that in the SBT ≥ 48 h group (33.6% vs. 25.3%; *p* <0.001, Table 1). Additionally, the LCx as the IRA was an independent predictor of all-cause death (*p* = 0.020, Table 4) in our study. Therefore, similar to STEMI, our study found that in patients with NSTEMI and pre-PCI TIMI 0/1, those who underwent PCI with a shorter SBT (<48 h) had a reduced duration of myocardial ischemia and lower mortality compared to those with a longer SBT (≥48 h). These findings suggest that shortening the SBT may confer a clinical benefit. Karwowski et al. [29] emphasized that although data are currently unavailable for NSTEMI patients with total occlusion, prompt reperfusion in the setting of complete blockage may lead to reduced infarct size and improved clinical outcomes. There is a discussion about the misconception of the term “STEMI”. While this term refers to a type of MI, it can make it difficult to accurately distinguish other forms of MI [30]. One notable example is the occlusion of the LCx, which may not be classified as STEMI. In the DIFOCCULT (Diagnostic accuracy of electrocardiogram for acute coronary occlusion resulting in myocardial infarction) study, the authors emphasized the importance of accurately diagnosing and differentiating LCx infarction by considering various factors, including electrocardiography findings and relevant clinical manifestations. Additionally, study [30] suggests that using more specific terms such as OMI (Occlusion MI) and Non-OMI may be helpful in clearly identifying the different forms of myocardial infarction. The LAD is responsible for supplying blood to 40–50% of the left ventricular myocardium, and blockages in this artery tend to result in larger infarcts, affecting 40% of the left ventricle compared with 18% for the RCA and 20% for the LCx [31,32]. In our study, as shown in Table S4, in the SBT < 48 h group, the pre-PCI TIMI 2/3 group exhibited higher LVEF and SBP compared to the pre-PCI TIMI 0/1 group, which may indicate improved coronary perfusion [33]. However, the pre-PCI TIMI 2/3 group had a higher percentage of the LAD as the IRA compared to the pre-PCI TIMI 0/1 group, and the presence of comorbidities such as hypertension, diabetes, and hyperlipidemia may have contributed to the relatively higher mortality rate. In addition, because recurrent MI is associated with increased long-term mortality [34], the increased incidence of recurrent MI in the pre-PCI TIMI 2/3 group compared to the pre-PCI TIMI 0/1 group may be linked to higher rates of all-cause death and CD in the pre-PCI TIMI 2/3 group. The significance of early reperfusion of the IRA to enhance clinical outcomes in patients with NSTEMI is less well defined when compared to STEMI patients [2]. According to meta-analysis [27], NSTEMI patients demonstrating complete occlusion of the IRA on CAG have a heightened mortality and major adverse cardiac events risk. In study [27], the authors stressed the need for more effective risk stratification tools capable of facilitating prompt revascularization, and potentially resulting in improved outcomes. Tziakas et al. [35] emphasized that a newly devised triage algorithm in NSTEMI patients is able to recognize those who resemble STEMI patients in terms of pathology and high-risk indicators. Such “STEMI equivalents” may derive potential benefits from an immediate invasive strategy. Generally, the absence of STE in patients with AMI is considered as evidence of incomplete coronary occlusion, leading to the conclusion that emergency myocardial reperfusion is not necessary [27]. Hence, it is possible that the SBT ≥ 48 h group within patients with pre-PCI TIMI 0/1 exhibits higher mortality rates than the SBT < 48 h group.

In the pre-PCI TIMI 0/1 group, SDT (<24 h) was a significant independent predictor of all-cause death (*p* = 0.033), whereas DBT (<24 h) was not (*p* = 0.461) (Table 4). This result is consistent with a recent report [12] that indicated that the presence of prehospital delay (SDT ≥ 24 h) increases the 3-year all-cause death in patients with NSTEMI (aHR, 1.35; *p* < 0.001). In study [12], DBT was not a significant prognostic factor for all-cause death. Recently, Meisel et al. [8] reported that SBT affects mortality in 4839 patients with STEMI, while DBT does not have an impact on mortality. The DBT interval coincides with the flat slope of the time–myonecrosis curve, where the effect of reperfusion on myocardial salvage is limited [8]. Hence, as previously mentioned, for patients with NSTEMI who present with pre-PCI TIMI 0/1, it is important to shorten the SBT, and reducing the SDT may have a greater impact than decreasing the DBT.

There is still ongoing debate regarding the optimal timing of appropriate reperfusion in patients with NSTEMI compared to those undergoing primary PCI for STEMI [10,11,12]. Additionally, there is a lack of research specifically focused on the pre-PCI TIMI flow grade in NSTEMI patients [3,4,26,27,28]. Our study is unique in that it is the first attempt to compare the impact of pre-PCI TIMI and SBT on the prognosis of patients with NSTEMI who underwent successful stent implantation. Our study establishes a significant association between a shorter SBT and lower 3-year mortality in NSTEMI patients with pre-PCI TIMI 0/1 flow compared to those with pre-PCI TIMI 2/3 flow. This study provides critical evidence that although predicting the pre-PCI TIMI 0/1 group using non-invasive modalities before CAG may be challenging [36], the presence of pre-PCI TIMI 0/1 flow after CAG indicates an unfavorable prognosis with higher future mortality than pre-PCI TIMI 2/3 flow. Consequently, in NSTEMI patients exhibiting pre-PCI TIMI 0/1 flow, it is essential to prioritize guideline-directed optimal medical treatment [9], close follow-up, and increased attention to reduce future mortality.

Despite the potential limitations associated with our sample size, the use of a registry derived from 20 high-volume tertiary university hospitals enabled us to provide important information about the relative importance of these two factors in determining long-term outcomes.

There were some drawbacks in this study. First, our study was hampered by a significant limitation: the inability to include several important variables in our analysis. These variables include transfer, distance to the nearest hospital, socioeconomic factors, and other potential barriers to rapid medical contact [37,38], because the primary factor leading to pre-hospital delay is the time it takes for individuals to interpret and respond to their symptoms. Moreover, medical care-seeking behavior has changed little over the past decades, even though numerous efforts have been made to educate the public about the detection of symptoms of MI and the benefits of immediate treatment [37,38]. The reason for this limitation was that these variables were not required for the KAMIR-NIH dataset, thereby restricting our ability to fully account for their potential influence on our findings. Second, the limited follow-up period of 3 years in our study could be viewed as a potential shortcoming, as it may not have been optimal for estimating long-term clinical outcomes. Third, a possible drawback of our study is that some subgroups had small sample sizes, which could have led to underpowered analyses and reduced our ability to detect significant differences that could have clinical relevance. Fourth, the KAMIR-NIH data used in our study may have contained underreported and/or missing data, which could represent a potential limitation of our findings. Fifth, our study is subject to a potential limitation in that variables not captured by the KAMIR-NIH study may have influenced our results, such as cognitive impairment, frailty, peripheral vasculopathy, type of medical insurance, history of cancer, symptom occurrence place, and the possibility of physician-generated selection bias in the treatment strategy, despite our efforts to conduct multivariate- and PS-adjusted analyses. These variables are important contributing factors to delayed hospitalization [12,37,38]. Sixth, the comparison of primary and secondary outcomes in our study was based on a 48 h cut-off point for SBT, which may represent a limitation. Different cut-off points could potentially yield different results, and the use of a single threshold may not capture the full complexity of SBT as a clinical phenomenon. Seventh, it should be noted that the lack of adjudication and analysis of TIMI flow grade by participating investigators is a crucial limitation of this study, despite the relatively low risk of misclassification resulting from the comparison of patients with (pre-TIMI 2/3) and without (pre-TIMI 0/1) patency [3]. Finally, the TIMI flow grade is a well-established technique for appraising coronary blood flow, primarily in situations involving acute coronary occlusion and/or reperfusion. Nevertheless, to obtain a more precise assessment, it is essential to take into account more informative benchmarks and relevant indicators, such as fractional flow reserve.

## 5. Conclusions

In conclusion, our findings indicate that shortening the SBT may improve survival outcomes in patients with NSTEMI and pre-PCI TIMI 0/1 flow. Therefore, for patients with NSTEMI and pre-PCI TIMI 0/1 who have an SBT longer than 48 h, it is necessary to identify the optimal approach for reducing mortality and achieving favorable outcomes. Additionally, conducting more studies using different SBT strategies and larger patient cohorts would be highly beneficial in the future.

## Figures and Tables

**Table 1:** Baseline characteristics.

Variables	Pre-PCI TIMI 0/1 (*n* = 1886)			Pre-PCI TIMI 2/3 (*n* = 3024)		
	SBT < 48 h (*n* = 1328, Group A)	SBT ≥ 48 h (*n* = 558, Group B)	*p* Value	SBT < 48 h (*n* = 1965, Group C)	SBT ≥ 48 h (*n* = 1059, Group D)	*p* Value
Male, *n* (%)	996 (75.5)	395 (70.8)	0.059	1436 (73.1)	723 (68.3)	0.006
Age, years	62.5 ± 12.2	66.3 ± 12.4	<0.001	64.4 ± 11.8	66.8 ± 12.0	<0.001
LVEF, %	53.4 ± 9.8	50.3 ± 9.4	<0.001	55.2 ± 10.9	54.1 ± 11.6	0.012
BMI, kg/m^2^	24.2 ± 3.4	23.9 ± 3.3	0.031	24.0 ± 3.3	24.1 ± 3.5	0.449
SBP, mmHg	133.7 ± 26.7	133.0 ± 28.0	0.609	136.0 ± 26.5	137.1 ± 24.6	0.256
DBP, mmHg	81.0 ± 16.4	79.7 ± 16.0	0.099	81.8 ± 15.4	81.3 ± 14.4	0.451
Cardiogenic shock, *n* (%)	71 (5.3)	19 (3.4)	0.076	96 (4.9)	34 (3.2)	0.031
CPR on admission, *n* (%)	39 (2.9)	21 (3.8)	0.388	64 (3.3)	35 (3.3)	0.944
SDT, hours	4.7 (2.0–11.3)	65.0 (21.5–114.2)	<0.001	4.0 (1.8–9.3)	48.0 (9.1–101.0)	<0.001
DBT, hours	6.2 (2.8–16.0)	24.6 (6.0–56.5)	<0.001	10.8 (3.6–19.9)	39.1 (16.7–63.2)	<0.001
Atypical chest pain, *n* (%)	178 (13.4)	137 (24.6)	<0.001	275 (14.0)	224 (21.2)	<0.001
Dyspnea, *n* (%)	297 (22.4)	202 (36.2)	<0.001	413 (21.0)	323 (30.5)	<0.001
EKG on admission						
Q-wave, *n* (%)	133 (10.0)	63 (11.3)	0.409	111 (5.6)	91 (8.6)	0.003
ST-segment depression, *n* (%)	308 (23.2)	112 (20.1)	0.146	465 (23.7)	225 (21.2)	0.134
T-wave inversion, *n* (%)	279 (21.0)	144 (25.8)	0.025	437 (22.2)	285 (26.9)	0.004
Atrial fibrillation, *n* (%)	48 (3.6)	25 (4.5)	0.363	86 (4.4)	49 (4.6)	0.782
Killip class 1I/III, *n* (%)	225 (16.9)	143 (25.6)	<0.001	249 (12.7)	182 (17.2)	0.001
First medical contact						
EMS, *n* (%)	167 (12.6)	31 (5.6)	<0.001	230 (11.7)	69 (6.5)	<0.001
Non-PCI center, *n* (%)	698 (52.6)	310 (55.6)	0.245	1047 (53.3)	574 (54.2)	0.647
PCI center, *n* (%)	463 (34.9)	217 (38.9)	0.103	688 (35.0)	416 (39.3)	0.022
Hypertension, *n* (%)	638 (48.0)	293 (52.5)	0.078	1077 (54.8)	617 (58.3)	0.071
Diabetes mellitus, *n* (%)	334 (25.2)	176 (31.5)	0.005	631 (32.1)	359 (33.9)	0.330
Dyslipidemia, *n* (%)	139 (10.5)	59 (10.6)	0.935	258 (13.1)	129 (12.2)	0.494
Previous MI, *n* (%)	97 (7.3)	33 (5.9)	0.319	132 (6.7)	81 (7.6)	0.371
Previous PCI, *n* (%)	132 (9.9)	48 (8.6)	0.391	196 (10.0)	108 (10.2)	0.849
Previous CABG, *n* (%)	6 (0.5)	5 (0.9)	0.319	19 (1.0)	10 (0.9)	0.951
Previous HF, *n* (%)	14 (1.1)	7 (1.3)	0.810	36 (1.8)	23 (2.2)	0.582
Previous stroke, *n* (%)	68 (5.1)	37 (6.6)	0.189	119 (6.1)	81 (7.6)	0.107
Current smokers, *n* (%)	530 (39.9)	175 (31.5)	0.001	732 (37.3)	332 (31.4)	0.001
Peak CK-MB, mg/dL	61.2 (11.5–153.9)	15.0 (5.0–51.8)	<0.001	19.8 (6.5–65.5)	10.8 (4.5–32.2)	0.005
Peak troponin-I, ng/mL	19.1 (4.8–42.7)	7.1 (2.2–20.8)	<0.001	5.4 (1.2–20.7)	3.0 (0.8–10.6)	<0.001
Serum creatinine (mg/L)	1.10 ± 1.28	1.23 ± 1.50	0.076	1.14 ± 1.37	1.18 ± 1.29	0.515
Total cholesterol, mg/dL	186.6 ± 46.8	179.5 ± 45.5	0.003	176.1 ± 45.1	173.5 ± 44.9	0.139
Triglyceride, mg/L	136.1 ± 125.2	128.2 ± 116.0	0.210	133.2 ± 137.4	128.85 ± 90.9	0.307
HDL cholesterol, mg/L	42.9 ± 11.5	42.0 ± 11.8	0.121	42.9 ± 11.7	42.3 ± 11.9	0.196
LDL cholesterol, mg/L	118.4 ± 39.8	112.5 ± 38.1	0.008	111.2 ± 38.5	109.0 ± 39.2	0.148
GRACE risk score	128.7 ± 42.4	139.2 ± 43.3	<0.001	130.9 ± 41.2	134.5 ± 38.8	0.017
Discharge medications, n (%)						
Aspirin, *n* (%)	1316 (99.1)	552 (98.9)	0.796	1942 (98.8)	1049 (99.1)	0.714
Clopidogrel, *n* (%)	951 (71.6)	428 (76.7)	0.023	1365 (69.5)	773 (73.0)	0.044
Ticagrelor, *n* (%)	239 (18.0)	82 (14.7)	0.093	418 (21.3)	207 (19.5)	0.279
Prasugrel, *n* (%)	138 (10.4)	48 (8.6)	0.271	182 (9.3)	79 (7.5)	0.103
BBs, *n* (%)	1132 (85.2)	456 (81.7)	0.062	1666 (84.8)	882 (83.3)	0.295
ACEI or ARBs, *n* (%)	1097 (82.6)	446 (79.9)	0.170	1648 (83.9)	879 (83.0)	0.538
Statin, *n* (%)	1251 (94.2)	515 (92.3)	0.122	1868 (95.1)	993 (93.8)	0.151
Anticoagulant, *n* (%)	38 (2.9)	22 (3.9)	0.250	43 (2.2)	33 (3.1)	0.143
Infarct-related artery						
Left main, *n* (%)	12 (0.9)	7 (1.3)	0.460	79 (4.0)	50 (4.7)	0.396
LAD, *n* (%)	410 (30.9)	195 (34.9)	0.084	966 (49.2)	505 (47.7)	0.446
LCx, *n* (%)	446 (33.6)	141 (25.3)	<0.001	450 (22.9)	235 (22.2)	0.682
RCA, *n* (%)	460 (34.6)	215 (38.5)	0.114	470 (23.9)	269 (25.4)	0.375
Treated vessel						
Left main, *n* (%)	28 (2.1)	20 (3.6)	0.077	106 (5.4)	69 (6.5)	0.221
LAD, *n* (%)	605 (45.6)	299 (53.6)	0.001	1227 (62.4)	668 (63.1)	0.753
LCx, *n* (%)	597 (45.0)	209 (37.5)	0.003	702 (35.7)	380 (35.9)	0.937
RCA, *n* (%)	565 (42.5)	257 (46.1)	0.170	651 (33.1)	372 (35.1)	0.277
ACC/AHA type B2/C lesions, *n* (%)	1148 (86.4)	493 (88.4)	0.293	1656 (84.3)	863 (81.5)	0.012
Transradial approach, *n* (%)	567 (42.7)	270 (48.4)	0.025	1111 (56.5)	597 (56.4)	0.939
GP IIb/IIIa inhibitor, *n* (%)	193 (14.5)	74 (13.3)	0.515	113 (5.8)	53 (5.0)	0.404
IVUS/OCT, *n* (%)	236 (17.8)	135 (24.2)	0.002	553 (28.1)	317 (29.9)	0.312
FFR, *n* (%)	15 (1.1)	10 (1.8)	0.272	44 (2.2)	36 (3.4)	0.074
Stents						
Bare-metal stent, *n* (%)	30 (2.3)	18 (3.2)	0.261	66 (3.4)	42 (4.0)	0.412
1st-generation DES, *n* (%)	43 (3.2)	13 (2.3)	0.372	79 (4.0)	52 (4.9)	0.262
2nd-generation DES, *n* (%)	1255 (94.5)	527 (94.4)	0.959	1820 (92.6)	965 (91.1)	0.158
Stent diameter (mm)	3.04 ± 0.42	3.02 ± 0.40	0.311	3.12 ± 0.44	3.09 ± 0.44	0.113
Stent length (mm)	30.4 ± 13.8	32.7 ± 16.2	0.003	28.0 ± 13.1	29.3 ± 14.3	0.011
Number of stents	1.21 ± 0.47	1.26 ± 0.51	0.063	1.18 ± 0.42	1.22 ± 0.48	0.008

Values are means ± standard deviation or median (interquartile range) or numbers and percentages. The *p* values for continuous data were obtained from the unpaired *t*-test. The *p* values for categorical data were obtained from the chi-square or Fisher’s exact test. Pre-PCI TIMI, pre-percutaneous coronary intervention thrombolysis in myocardial infarction flow grade; SBT, symptom-to-balloon time; LVEF, left ventricular ejection fraction; BMI, body mass index; SBP, systolic blood pressure; DBP, diastolic blood pressure; CPR, cardiopulmonary resuscitation; SDT, symptom-to-door time; DBT, door-to-balloon time; EKG, electrocardiogram; EMS, emergency medical service; MI, myocardial infarction; CABG, coronary artery bypass graft; HF, heart failure; CK-MB, creatine kinase myocardial band; HDL, high-density lipoprotein; LDL, low-density lipoprotein; GRACE, Global Registry of Acute Coronary Events; BBs, beta-blockers; ACEIs, angiotensin converting enzyme inhibitors; ARBs, angiotensin receptor blockers; LAD, left anterior descending coronary artery; LCx, left circumflex coronary artery; RCA, right coronary artery; ACC/AHA, American College of Cardiology/American Heart Association; GP, glycoprotein; IVUS/OCT, intravascular ultrasound/optical coherence tomography; FFR, fractional flow reserve; DES, drug-eluting stent.

**Table 2:** Clinical outcomes between the SBT < 48 h and SBT ≥ 48 h groups in patients with pre-PCI TIMI 0/1 or TIMI 2/3 at 3 years.

**Outcomes**	**Pre-PCI TIMI 0/1, *n* = 1886**								
	**SBT < 48 h** **(*n* = 1328, Group A)**	**SBT ≥ 48 h** **(*n* = 558, Group B)**	**Log-Rank**	**Unadjusted**		**Multivariable-Adjusted ^a^**		**Propensity Score-Adjusted**	
				**HR (95% CI)**	***p***	**HR (95% CI)**	***p***	**HR (95% CI)**	***p***
All-cause death	97 (7.3)	62 (11.1)	0.007	0.646 (0.470–0.889)	0.007	1.877 (1.230–2.865)	0.003	1.932 (0.277–2.924)	0.002
Cardiac death	50 (3.8)	44 (7.9)	<0.001	0.470 (0.313–0.705)	<0.001	2.648 (1.582–4.433)	<0.001	2.712 (1.614–4.545)	<0.001
Non-cardiac death	47 (3.5)	18 (3.2)	0.791	1.076 (0.625–1.853)	0.791	1.062 (0.472–2.392)	0.884	1.166 (0.530–2.656)	0.702
Recurrent MI	29 (2.3)	20 (3.8)	0.072	0.596 (0.337–1.054)	0.075	1.276 (0.626–2.600)	0.502	1.359 (0.672–2.745)	0.393
Any repeat revascularization	107 (8.4)	49 (9.4)	0.563	0.905 (0.645–1.269)	0.563	1.396 (0.863–2.259)	0.174	1.298 (0.801–2.100)	0.271
All-cause death, recurrent MI, or any repeat revascularization	205 (15.4)	104 (18.6)	0.098	0.820 (0.674–1.038)	0.099	1.147 (1.035–1.941)	0.030	1.139 (1.030–1.938)	0.032
**Outcomes**	**Pre-PCI TIMI 2/3, *n* = 3024**								
	**SBT < 48 h** **(*n* = 1965, Group C)**	**SBT ≥ 48 h** **(*n* = 1059, Group D)**	**Log-Rank**	**Unadjusted**		**Multivariable-Adjusted ^a^**		**Propensity Score-Adjusted**	
				**HR (95% CI)**	***p***	**HR (95% CI)**	***p***	**HR (95% CI)**	***p***
All-cause death	191 (9.7)	108 (10.2)	0.684	0.952 (0.752–1.206)	0.684	1.108 (0.820–1.497)	0.503	1.082 (0.799–1.450)	0.600
Cardiac death	119 (6.1)	62 (5.8)	0.834	1.033 (0.760–1.405)	0.834	1.034 (0.705–1.517)	0.863	1.027 (0.697–1.487)	0.895
Non-cardiac death	72 (3.6)	46 (4.4)	0.363	0.843 (0.582–1.220)	0.364	1.405 (0.854–2.310)	0.180	1.323 (0.820–2.135)	0.251
Recurrent MI	76 (4.1)	41 (4.0)	0.990	1.002 (0.685–1.465)	0.990	1.139 (0.696–1.863)	0.606	1.201 (0.723–1.903)	0.554
Any repeat revascularization	178 (9.6)	83 (8.3)	0.249	1.165 (0.898–1.512)	0.250	1.093 (0.771–1.549)	0.618	1.103 (0.794–1.571)	0.560
All-cause death, recurrent MI, or any repeat revascularization	368 (18.7)	188 (17.8)	0.499	1.062 (0.891–1.266)	0.499	1.018 (0.808–1.281)	0.882	1.031 (0.823–1.291)	0.791
**Outcomes**		**Total, *n* = 4910**							
	**SBT < 48 h** **(*n* = 3293, Group** **A + C)**	**SBT ≥ 48 h** **(*n* = 1617, Group** **B + D)**	**Log-Rank**	**Unadjusted**		**Multivariable-Adjusted ^a^**		**Propensity Score-Adjusted**	
				**HR (95% CI)**	***p***	**HR (95% CI)**	***p***	**HR (95% CI)**	***p***
All-cause death	288 (8.7)	170 (10.5)	0.047	0.826 (0.683–0.998)	0.048	1.278 (1.001–1.630)	0.047	1.291 (1.011–1.720)	0.040
Cardiac death	169 (5.1)	106 (6.5)	0.042	0.778 (0.610–0.991)	0.042	1.340 (0.987–1.819)	0.060	1.354 (1.007–1.828)	0.050
Non-cardiac death	119 (3.6)	64 (4.0)	0.522	0.899 (0.688–1.227)	0.522	1.209 (0.802–1.825)	0.365	1.217 (0.819–1.932)	0.331
Recurrent MI	105 (3.4)	61 (3.8)	0.298	0.837 (0.611–1.148)	0.298	1.183 (0.788–1.778)	0.418	1.214 (0.812–1.815)	0.344
Any repeat revascularization	285 (9.1)	132 (8.7)	0.579	1.060 (0.863–1.303)	0.579	1.013 (0.765–1.340)	0.930	1.009 (0.624–1.217)	0.953
All-cause death, recurrent MI, or any repeat revascularization	573 (17.4)	292 (18.1)	0.609	0.964 (0.837–1.110)	0.609	1.086 (0.903–1.306)	0.382	1.072 (0.892–1.298)	0.361

SBT, symptom-to-balloon time; pre-PCI TIMI, pre-percutaneous coronary intervention thrombolysis in myocardial infarction flow grade; HR, hazard ratio; CI, confidence interval; MI, myocardial infarction; LVEF, left ventricular ejection fraction; BMI, body mass index: CPR, cardiopulmonary resuscitation; SDT, symptom-to-door time; DBT, door-to-balloon time; EKG, electrocardiogram; EMS, emergency medical service; DM, diabetes mellitus; CK-MB, creatine kinase myocardial band; LDL, low-density lipoprotein; GRACE, Global Registry of Acute Coronary Events. ^a^ adjusted by male sex, age, LVEF, BMI, cardiogenic shock, CPR on admission, SDT, DBT, atypical chest pain, dyspnea, Q-wave, T-wave inversion on EKG, Killip class II/III, EMS, PCI center, DM, current smoker; peak CK-MB, peak troponin-I, total cholesterol, LDL-cholesterol, GRACE risk score, and clopidogrel (Table S1).

**Table 3:** Clinical outcomes in the pre-PCI TIMI 0/1 and pre-PCI TIMI 2/3 groups in patients with SBT < 48 h or ≥48 h at 3 years.

**Outcomes**	**SBT < 48 h, *n* = 3293**						
	**Pre-PCI TIMI 0/1** **(*n* = 1328,** **Group A)**	**Pre-PCI TIMI 2/3** **(*n* = 1965,** **Group C)**	**Log-Rank**	**Unadjusted**		**Multivariable-Adjusted ^a^**	
				**HR (95% CI)**	***p***	**HR (95% CI)**	***p***
All-cause death	97 (7.3)	191 (9.7)	0.019	0.747 (0.585–0.954)	0.019	1.347 (1.021–1.778)	0.035
Cardiac death	50 (3.8)	119 (6.1)	0.004	0.613 (0.444–0.860)	0.004	1.491 (1.032–2.156)	0.034
Non-cardiac death	47 (3.5)	72 (3.6)	0.825	0.959 (0.664–1.386)	0.825	1.166 (0.764–1.779)	0.477
Recurrent MI	29 (2.3)	76 (4.1)	0.007	0.557 (0.363–0.855)	0.007	1.740 (1.098–2.758)	0.018
Any repeat revascularization	107 (8.4)	178 (9.6)	0.326	0.887 (0.698–1.127)	0.326	1.147 (0.872–1.509)	0.331
All-cause death, recurrent MI, or any repeat revascularization	205 (15.4)	368 (18.7)	0.023	0.821 (0.692–0.974)	0.024	1.308 (1.078–1.587)	0.007
**Outcomes**	**SBT ≥ 48 h, *n* = 1617**						
	**Pre-PCI TIMI 0/1** **(*n* = 558, Group B)**	**Pre-PCI TIMI 2/3** **(*n* = 1059, Group D)**	**Log-Rank**	**Unadjusted**		**Multivariable-Adjusted ^a^**	
				**HR (95% CI)**	***p***	**HR (95% CI)**	***p***
All-cause death	62 (11.1)	108 (10.2)	0.539	1.103 (0.807–1.507)	0.539	1.040 (0.740–1.461)	0.822
Cardiac death	44 (7.9)	62 (5.8)	0.117	1.361 (0.925–2.002)	0.118	1.210 (0.797–1.837)	0.370
Non-cardiac death	18 (3.2)	46 (4.4)	0.309	0.754 (0.437–1.301)	0.311	1.559 (0.848–2.866)	0.152
Recurrent MI	20 (3.8)	41 (4.0)	0.806	0.935 (0.548–1.596)	0.806	1.136 (0.637–2.026)	0.667
Any repeat revascularization	49 (9.4)	83 (8.3)	0.460	1.142 (0.802–1.626)	0.460	1.099 (0.740–1.631)	0.641
All-cause death, recurrent MI, or any repeat revascularization	104 (18.6)	188 (17.8)	0.609	1.064 (0.838–1.353)	0.609	1.042 (0.799–1.358)	0.762
**Outcomes**	**Total, *n* = 4910**						
	**Pre-PCI TIMI 0/1** **(*n*= 1886, Group A + B)**	**Pre-PCI TIMI 2/3** **(*n* = 3024, Group C + D)**	**Log-Rank**	**Unadjusted**		**Multivariable-Adjusted ^a^**	
				**HR (95% CI)**	***p***	**HR (95% CI)**	***p***
All-cause death	159 (8.4)	299 (9.9)	0.102	0.852 (0.703–1.033)	0.102	1.234 (0.999–1.525)	0.052
Cardiac death	94 (5.0)	181 (6.0)	0.147	0.832 (0.648–1.067)	0.148	1.198 (0.914–1.570)	0.191
Non-cardiac death	65 (3.4)	118 (3.9)	0.418	0.883 (0.652–1.195)	0.418	1.300 (0.924–1.829)	0.133
Recurrent MI	49 (2.7)	117 (4.1)	0.017	0.668 (0.478–0.932)	0.018	1.486 (1.039–2.125)	0.030
Any repeat revascularization	156 (8.7)	261 (9.1)	0.701	0.962 (0.789–1.173)	0.701	1.054 (0.842–1.319)	0.646
All-cause death, recurrent MI, or any repeat revascularization	309 (16.4)	556 (18.4)	0.109	0.893 (0.777–1.026)	0.109	1.200 (1.028–1.401)	0.020

Pre-PCI TIMI, pre-percutaneous coronary intervention thrombolysis in myocardial infarction flow grade; SBT, symptom-to-balloon time; HR, hazard ratio; CI, confidence interval; MI, myocardial infarction; LVEF, left ventricular ejection fraction; BMI, body mass index: SBP, systolic blood pressure; DBP, diastolic blood pressure; CPR, cardiopulmonary resuscitation; SDT, symptom-to-door time; DBT, door-to-balloon time; DM, diabetes mellitus; CK-MB, creatine kinase myocardial band; LDL, low-density lipoprotein; GRACE, Global Registry of Acute Coronary Events; IRA, infarct-related artery; LAD, left anterior descending artery; LCx, left circumflex artery. ^a^ adjusted by male sex, age, LVEF, BMI, SBP, DBP, cardiogenic shock, CPR on admission, SDT, DBT, atypical chest pain, dyspnea, Q-wave, Killip class II/III, hypertension, DM, dyslipidemia, peak CK-MB, peak troponin-I, total cholesterol, LDL-cholesterol, GRACE risk score, ticagrelor, left main (IRA), LAD (IRA), and LCx (IRA) (Table S3).

**Table 4:** Independent predictors for all-cause death.

Variables	Pre-PCI TIMI 0/1				Pre-PCI TIMI 2/3			
	Unadjusted		Adjusted ^a^		Unadjusted		Adjusted ^a^	
	HR (95% CI)	*p* Value	HR (95% CI)	*p* Value	HR (95% CI)	*p* Value	HR (95% CI)	*p* Value
SBT < 48 h vs. SBT ≥ 48 h	0.646 (0.470–0.889)	0.007	0.567 (0.425–0.864)	0.005	0.952 (0.752–1.206)	0.684	1.075 (0.722–1.398)	0.522
Male	0.528 (0.384–0.726)	<0.001	1.100 (0.757–1.600)	0.617	0.617 (0.489–0.779)	<0.001	1.259 (0.951–1.667)	0.108
Age, ≥65 years	4.981 (3.331–7.450)	<0.001	2.178 (1.325–3.579)	0.002	5.419 (3.923–7.486)	<0.001	2.318 (1.580–3.402)	<0.001
LVEF, <50%	4.231 (3.038–5.894)	<0.001	1.625 (1.122–2.353)	0.010	3.570 (2.843–4.483)	<0.001	1.594 (1.206–2.108)	0.002
Cardiogenic shock	5.211 (3.465–7.838)	<0.001	1.649 (0.922–2.949)	0.092	6.328 (4.680–8.555)	<0.001	2.409 (1.633–3.554)	<0.001
CPR on admission	12.64 (8.607–18.57)	<0.001	4.242 (2.382–7.554)	<0.001	9.123 (6.712–12.40)	<0.001	2.183 (1.439–3.311)	<0.001
SDT <24 h	1.809 (1.317–2.483)	<0.001	1.496 (1.033–2.167)	0.033	1.245 (0.978–1.586)	0.075	1.044 (0.790–1.308)	0.362
DBT <24 h	1.028 (0.706–1.495)	0.887	1.180 (0.760–1.830)	0.461	1.105 (0.860–1.421)	0.436	1.073 (0.815–1.411)	0.517
Atypical chest pain	4.432 (3.242–6.060)	<0.001	2.349 (1.617–3.414)	<0.001	3.734 (2.962–4.705)	<0.001	1.587 (1.176–2.140)	0.003
Dyspnea	2.054 (1.498–2.816)	<0.001	1.058 (0.718–1.557)	0.776	2.243 (1.780–2.826)	<0.001	1.051 (0.775–1.427)	0.748
EMS (+)	1.244 (0.779–1.988)	0.361	1.475 (0.878–2.480)	0.142	1.276 (0.901–1.807)	0.171	1.164 (0.782–1.731)	0.455
Hypertension	1.733 (1.257–2.388)	0.001	1.028 (0.708–1.493)	0.885	1.546 (1.218–1.964)	<0.001	1.064 (0.799–1.418)	0.671
Diabetes mellitus	1.831 (1.332–2.515)	<0.001	1.381 (1.001–1.796)	0.101	1.822 (1.451–2.287)	<0.001	1.459 (1.110–1.917)	0.013
LCx (IRA)	1.495 (1.104–2.026)	0.009	1.421 (1.139–2.017)	0.020	1.050 (0.749–1.473)	0.777	1.176 (0.843–1.642)	0.340
GRACE risk score >140	7.472 (5.093–10.96)	<0.001	2.513 (1.513–4.174)	<0.001	6.816 (4.756–8.044)	<0.001	2.200 (1.552–3.120)	<0.001

Pre-PCI TIMI, pre-percutaneous coronary intervention thrombolysis in myocardial infarction flow grade; HR, hazard ratio; CI, confidence interval; SBT, symptom-to-balloon time; LVEF, left ventricular ejection fraction; CPR, cardiopulmonary resuscitation; SDT, symptom-to-door time; DBT, door-to-balloon time; EMS, emergency medical service; LCx, left circumflex coronary artery; IRA, infarct-related artery; GRACE, Global Registry of Acute Coronary Events. ^a^ adjusted by male sex, age, LVEF, BMI, cardiogenic shock, CPR on admission, SDT, DBT, atypical chest pain, dyspnea, Q-wave, T-wave inversion on EKG, Killip class II/III, EMS, PCI center, DM, current smoker; peak CK-MB, peak troponin-I, total cholesterol, LDL-cholesterol, GRACE risk score, and clopidogrel (Table S1).

## Data Availability

Data are contained within the article or Supplementary Materials.

## Supplementary Materials

The following supporting information can be downloaded at: https://www.mdpi.com/article/10.3390/jcm12113654/s1. Table S1: Results of the collinearity test for all-cause death between the SBT < 48 h and SBT ≥ 48 h groups, Table S2: Baseline characteristics between the SBT < 48 h and SBT ≥ 48 h groups before and after propensity score-matched analysis, Table S3: Results of the collinearity test for all-cause death between the pre-PCI TIMI 0/1 and pre-PCI TIMI 2/3 groups, Table S4: Baseline characteristics between the pre-PCI TIMI 0/1 and pre-PCI TIMI 2/3 groups in patients with SBT < 48 h or ≥48 h.
