# Supplementary material for: Three-Year Clinical Outcomes Based on Pre-Percutaneous Coronary Intervention Coronary Blood Flow Grade and Symptom-to-Balloon Time in Patients with Non-ST-Segment Elevation Myocardial Infarction
Source: J Clin Med. 2023 May 24;12(11):3654. doi: 10.3390/jcm12113654 (PMC10254009; doi:10.3390/jcm12113654)
Supplement: Supplementary file 1 [file jcm-12-03654-s001.zip › jcm-2409913-supplementary.pdf]

# Supplementary Materials

# Three-year clinical outcomes based on pre-percutaneous coronary intervention coronary blood flow grade and symptom-to-balloon time in patients with non-ST-segment elevation myocardial infarction

## Supplementary Online Contents


**Table S1.** Results of the collinearity test for all-cause death between the SBT <48 h and SBT ≥48 h groups

**Table S2.** Baseline characteristics between the SBT <48 h and SBT ≥48 h groups before and after propensity score-matched analysis

**Table S3.** Results of the collinearity test for all-cause death between the pre-PCI TIMI 0/1 and pre-PCI TIMI 2/3 groups

**Table S4.** Baseline characteristics between the pre-PCI TIMI 0/1 and pre-PCI TIMI 2/3 groups in patients with SBT <48 h or ≥48 h


**Table S1.** Results of the collinearity test for all-cause death between the SBT <48 h and SBT ≥48 h groups

| | Variance Inflation Factors | Tolerance | Condition Index |
|---|---|---|---|
| Male | 1.308 | 0.765 | 1.000 |
| Age | 2.438 | 0.410 | 3.036 |
| LVEF | 1.310 | 0.763 | 3.122 |
| BMI | 1.173 | 0.852 | 3.527 |
| Cardiogenic shock | 1.315 | 0.761 | 3.881 |
| CPR on admission | 1.513 | 0.661 | 3.928 |
| SDT | 1.041 | 0.960 | 4.135 |
| DBT | 1.080 | 0.926 | 4.352 |
| Atypical chest pain | 1.195 | 0.837 | 4.453 |
| Dyspnea | 1.244 | 0.804 | 4.545 |
| Q-wave | 1.033 | 0.968 | 4.757 |
| T-wave inversion | 1.102 | 0.907 | 4.843 |
| Killip class II/III | 1.515 | 0.660 | 5.166 |
| EMS | 1.089 | 0.919 | 5.205 |
| PCI center | 1.120 | 0.893 | 5.349 |
| Diabetes mellitus | 1.109 | 0.902 | 5.412 |
| Current smoker | 1.326 | 0.754 | 5.673 |
| Peak CK-MB | 1.193 | 0.838 | 5.787 |

| | | | |
|---|---|---|---|
| Peak troponin-I | 1.229 | 0.814 | 5.944 |
| Total cholesterol | 4.291 | 0.233 | 6.312 |
| LDL-cholesterol | 4.229 | 0.236 | 7.493 |
| GRACE risk score | 3.057 | 0.327 | 8.567 |
| Clopidogrel | 1.097 | 0.912 | 9.252 |
| LCx (IRA) | 3.384 | 0.296 | 10.695 |
| LAD (treated vessel) | 2.192 | 0.456 | 12.402 |
| LCx (treated vessel) | 2.203 | 0.454 | 14.510 |
| ACC/AHA type B2/C lesions | 1.082 | 0.924 | 22.085 |
| Transradial approach | 1.104 | 0.906 | 27.883 |
| IVUS/OCT | 1.063 | 0.941 | 36.427 |
| Stent length | 2.523 | 0.396 | 40.641 |
| Number of stents | 2.389 | 0.418 | 68.321 |

SBT, symptom-to-balloon time; LVEF, left ventricular ejection fraction; BMI, body mass index; CPR, cardiopulmonary resuscitation; SDT, symptom-to-door time; DBT, door-to-balloon time; EMS, emergency medical service; PCI, percutaneous coronary intervention; CK-MB, creatine kinase myocardial band; LDL, low-density lipoprotein; GRACE, Global Registry of Acute Coronary Events; LCx, left circumflex coronary artery; IRA, infarct-related artery; LAD, left anterior descending coronary artery; ACC/AHA, American College of Cardiology/American Heart Association; IVUS, intravascular ultrasound; OCT, optical coherence tomography.

**Table S2.** Baseline characteristics between the SBT <48 h and SBT ≥48 h groups before and after propensity score-matched analysis

| Variables | All patients (n = 4910) | | | Propensity score-matched patients (n = 3058) | | | |
|---|---|---|---|---|---|---|---|
| | SBT <48 h (*n* = 3293, group A+C) | SBT ≥48 h (*n* = 1617, group B+D) | *p* value | SBT <48 h (*n* = 1529) | SBT ≥48 h (*n* = 1529) | *p* value | SD |
| Male, *n* (%) | 2432 (73.9) | 1118 (69.1) | 0.001 | 1073 (70.2) | 1071 (70.0) | 0.968 | 0.04 |
| Age, years | 63.6 ± 12.0 | 66.6 ± 12.1 | <0.001 | 66.2 ± 11.6 | 66.2 ± 12.2 | 0.881 | -0.05 |
| LVEF, % | 54.5 ± 10.5 | 52.8 ± 11.9 | <0.001 | 53.4 ± 11.1 | 53.3 ± 11.6 | 0.851 | 0.09 |
| BMI, kg/m$^2$ | 24.1 ± 3.3 | 24.0 ± 3.4 | 0.462 | 24.1 ± 3.3 | 24.0 ± 3.4 | 0.517 | 0.29 |
| SBP, mmHg | 135.1 ± 26.9 | 135.7 ± 25.6 | 0.437 | 135.6 ± 26.4 | 135.7 ± 25.7 | 0.878 | -0.03 |
| DBP, mmHg | 81.5 ± 15.8 | 80.8 ± 15.0 | 0.133 | 80.8 ± 15.5 | 80.9 ± 15.1 | 0.784 | -0.07 |
| Cardiogenic shock, *n* (%) | 167 (5.1) | 53 (3.3) | 0.004 | 60 (3.9) | 52 (3.4) | 0.501 | 0.27 |
| CPR on admission, *n* (%) | 103 (3.1) | 56 (3.5) | 0.549 | 53 (3.5) | 52 (3.4) | 0.921 | 0.05 |
| Atypical chest pain, *n* (%) | 453 (13.8) | 361 (22.3) | <0.001 | 291 (19.0) | 305 (19.9) | 0.553 | -0.23 |
| Dyspnea, n (%) | 710 (21.6) | 525 (32.5) | <0.001 | 459 (30.0) | 455 (29.8) | 0.906 | 0.04 |
| EKG on admission | | | | | | | |
| Q-wave, n (%) | 244 (7.4) | 154 (9.5) | 0.012 | 134 (8.8) | 133 (8.7) | 0.949 | 0.03 |
| ST-segment depression, *n* (%) | 773 (23.5) | 337 (20.8) | 0.039 | 345 (22.6) | 325 (21.3) | 0.406 | 0.31 |
| T-wave inversion, *n* (%) | 716 (21.7) | 429 (26.5) | <0.001 | 406 (26.6) | 400 (26.2) | 0.837 | 0.09 |
| Atrial fibrillation, *n* (%) | 134 (4.1) | 74 (4.6) | 0.408 | 64 (4.2) | 69 (4.5) | 0.723 | -0.15 |
| Killip class 1I/III, *n* (%) | 474 (14.4) | 325 (20.1) | <0.001 | 289 (18.9) | 286 (18.7) | 0.926 | 0.05 |
| First medical contact | | | | | | | |
| EMS, *n* (%) | 397 (12.1) | 100 (6.2) | <0.001 | 99 (6.5) | 100 (6.5) | 0.942 | -0.03 |
| Non-PCI center, *n* (%) | 1745 (53.0) | 884 (54.7) | 0.273 | 845 (55.3) | 837 (54.7) | 0.799 | 0.12 |
| PCI center, *n* (%) | 1151 (35.0) | 633 (39.1) | 0.004 | 585 (38.3) | 592 (38.7) | 0.824 | -0.08 |
| Hypertension, *n* (%) | 1715 (52.1) | 910 (56.3) | 0.006 | 874 (57.2) | 857 (56.0) | 0.559 | 0.24 |
| Diabetes mellitus, *n* (%) | 965 (29.3) | 535 (33.1) | 0.008 | 511 (33.4) | 497 (32.5) | 0.617 | 0.19 |
| Dyslipidemia, *n* (%) | 397 (12.1) | 188 (11.6) | 0.673 | 175 (11.4) | 180 (11.8) | 0.821 | -0.12 |
| Previous MI, *n* (%) | 229 (7.0) | 114 (7.1) | 0.905 | 111 (7.3) | 107 (7.0) | 0.833 | 0.12 |
| Previous PCI, *n* (%) | 328 (10.0) | 156 (9.6) | 0.760 | 153 (10.0) | 150 (9.8) | 0.904 | 0.07 |

| | | | | | | | |
|---|---|---|---|---|---|---|---|
| Previous CABG, *n* (%) | 25 (0.8) | 15 (0.9) | 0.613 | 15 (1.0) | 14 (0.9) | 0.852 | 0.10 |
| Previous HF, *n* (%) | 50 (1.5) | 30 (1.9) | 0.402 | 27 (1.8) | 28 (1.8) | 0.892 | -0.09 |
| Previous stroke, *n* (%) | 187 (5.7) | 118 (7.3) | 0.032 | 112 (7.3) | 108 (7.1) | 0.834 | 0.08 |
| Current smokers, *n* (%) | 1262 (38.3) | 508 (31.4) | <0.001 | 498 (32.6) | 494 (32.3) | 0.908 | 0.06 |
| Peak CK-MB, mg/dL | 29.6 (7.7-99.9) | 12.3 (4.8-42.3) | <0.001 | 17.3 (5.7-56.9) | 12.9 (4.9-44.8) | 0.474 | 0.40 |
| Peak troponin-I, ng/mL | 13.0 (2.5-24.0) | 5.0 (1.3-21.6) | <0.001 | 6.7 (1.6-22.5) | 5.2 (1.3-22.5) | 0.200 | 0.46 |
| Serum creatinine (mg/L) | 1.13 ± 1.33 | 1.20 ± 1.37 | 0.098 | 1.18 ± 1.39 | 1.18 ± 1.37 | 0.979 | 0.01 |
| Total cholesterol, mg/dL | 180.3 ± 46.1 | 175.6 ± 45.2 | 0.001 | 176.1 ± 45.6 | 176.1 ± 44.6 | 0.974 | -0.01 |
| Triglyceride, mg/L | 134.4 ± 112.6 | 128.6 ± 100.4 | 0.102 | 131.4 ± 99.2 | 129.8 ± 98.3 | 0.693 | 0.16 |
| HDL cholesterol, mg/L | 42.9 ± 11.6 | 42.2 ± 11.9 | 0.048 | 41.7 ± 10.9 | 42.3 ± 11.4 | 0.165 | -0.54 |
| LDL cholesterol, mg/L | 113.8 ± 39.1 | 110.0 ± 38.9 | 0.003 | 111.1 ± 37.3 | 110.6 ± 36.6 | 0.712 | 0.13 |
| GRACE risk score | 130.0 ± 41.7 | 136.1 ± 40.5 | <0.001 | 135.4 ± 41.7 | 135.0 ± 40.5 | 0.796 | 0.09 |
| Discharge medications, *n* (%) | | | | | | | |
| Aspirin, *n* (%) | 3258 (98.9) | 1601 (99.0) | 0.882 | 1512 (98.9) | 1513 (99.0) | 0.861 | -0.10 |
| Clopidogrel, *n* (%) | 2316 (70.3) | 1201 (74.3) | 0.004 | 1139 (74.5) | 1130 (73.9) | 0.741 | 0.14 |
| Ticagrelor, *n* (%) | 657 (20.0) | 289 (17.9) | 0.083 | 270 (17.7) | 278 (18.2) | 0.741 | -0.13 |
| Prasugrel, *n* (%) | 320 (9.7) | 127 (7.9) | 0.035 | 120 (7.8) | 121 (7.9) | 0.946 | -0.04 |
| BBs, *n* (%) | 2798 (85.0) | 1338 (82.7) | 0.046 | 1279 (83.6) | 1273 (83.3) | 0.808 | 0.08 |
| ACEI or ARBs, *n* (%) | 2745 (83.4) | 1282 (79.3) | 0.001 | 1230 (80.4) | 1226 (80.2) | 0.891 | 0.05 |
| Statin, *n* (%) | 3119 (94.7) | 1508 (93.3) | 0.043 | 1435 (93.9) | 1431 (93.6) | 0.823 | 0.12 |
| Anticoagulant, *n* (%) | 56 (1.7) | 50 (3.1) | 0.002 | 45 (2.9) | 39 (2.6) | 0.580 | 0.18 |
| Infarct-related artery | | | | | | | |
| Left main, *n* (%) | 91 (2.8) | 57 (3.5) | 0.155 | 49 (3.2) | 53 (3.5) | 0.689 | -0.17 |
| LAD, *n* (%) | 1376 (41.8) | 700 (43.3) | 0.325 | 674 (44.1) | 658 (43.0) | 0.584 | 0.22 |
| LCx, *n* (%) | 896 (27.2) | 363 (22.4) | <0.001 | 346 (22.6) | 354 (23.2) | 0.763 | -0.14 |
| RCA, *n* (%) | 930 (28.2) | 484 (29.9) | 0.227 | 460 (30.1) | 464 (30.3) | 0.906 | -0.04 |
| Treated vessel | | | | | | | |
| Left main, *n* (%) | 134 (4.1) | 89 (5.5) | 0.028 | 81 (5.3) | 78 (5.1) | 0.871 | 0.09 |
| LAD, *n* (%) | 1832 (55.6) | 967 (59.8) | 0.006 | 911 (59.6) | 913 (59.7) | 0.971 | -0.02 |
| LCx, *n* (%) | 1299 (39.4) | 589 (36.4) | 0.042 | 563 (36.8) | 564 (36.9) | 0.970 | -0.02 |

| | | | | | | | |
|---|---|---|---|---|---|---|---|
| RCA, *n* (%) | 1216 (36.9) | 629 (38.9) | 0.188 | 593 (38.8) | 604 (39.5) | 0.711 | -0.14 |
| ACC/AHA type B2/C lesions, *n* (%) | 2804 (85.2) | 1356 (84.0) | 0.310 | 1291 (84.4) | 1288 (84.2) | 0.921 | 0.05 |
| Transradial approach, *n* (%) | 1678 (51.0) | 867 (53.6) | 0.083 | 798 (52.2) | 816 (53.4) | 0.538 | -0.24 |
| GP IIb/IIIa inhibitor, *n* (%) | 306 (9.3) | 127 (7.9) | 0.097 | 116 (7.6) | 114 (7.5) | 0.945 | 0.04 |
| IVUS/OCT, *n* (%) | 789 (24.0) | 452 (28.0) | 0.003 | 426 (27.9) | 414 (27.1) | 0.656 | 0.18 |
| FFR, *n* (%) | 59 (1.8) | 46 (2.8) | 0.021 | 45 (2.9) | 44 (2.9) | 0.914 | 0.04 |
| Stent | | | | | | | |
| Bare-metal stent, n (%) | 96 (2.9) | 60 (3.7) | 0.141 | 50 (3.3) | 56 (3.7) | 0.621 | -0.22 |
| 1$^{st}$-generation DES, n (%) | 122 (3.7) | 65 (4.0) | 0.580 | 61 (4.0) | 59 (3.9) | 0.926 | 0.05 |
| 2$^{nd}$-generation DES, n (%) | 3075 (93.4) | 1492 (92.3) | 0.153 | 1418 (92.7) | 1414 (92.5) | 0.836 | 0.07 |
| Stent diameter (mm) | 3.08 ± 0.44 | 3.06 ± 0.43 | 0.121 | 3.06 ± 0.42 | 3.07 ± 0.43 | 0.794 | -0.23 |
| Stent length (mm) | 28.9 ± 13.4 | 30.5 ± 15.1 | <0.001 | 30.3 ± 14.1 | 30.2 ± 14.8 | 0.923 | 0.06 |
| Number of stents | 1.19 ± 0.44 | 1.24 ± 0.49 | 0.002 | 1.23 ± 0.49 | 1.23 ± 0.48 | 0.765 | 0.24 |

Values are means ± standard deviation or median (interquartile range) or numbers and percentages. The *p* values for continuous data were obtained from the unpaired t-test. The *p* values for categorical data were obtained from the chi-square or Fisher’s exact test. Pre-PCI TIMI, pre-percutaneous coronary intervention thrombolysis in myocardial infarction flow grade; SBT, symptom-to-balloon time; LVEF, left ventricular ejection fraction; BMI, body mass index; SBP, systolic blood pressure; DBP, diastolic blood pressure; CPR, cardiopulmonary resuscitation; EKG, electrocardiogram; EMS, emergency medical service; MI, myocardial infarction; CABG, coronary artery bypass graft; HF, heart failure; CK-MB, creatine kinase myocardial band; HDL, high-density lipoprotein; LDL, low-density lipoprotein; GRACE, Global Registry of Acute Coronary Events; BBs, beta-blockers; ACEIs, angiotensin converting enzyme inhibitors; ARBs, angiotensin receptor blockers; LAD, left anterior descending coronary artery; LCx, left circumflex coronary artery; RCA, right coronary artery; ACC/AHA, American College of Cardiology/American Heart Association; GP, glycoprotein; IVUS/OCT, intravascular ultrasound/optical coherence tomography; FFR, fractional flow reserve; DES, drug-eluting stent.

**Table S3.** Results of the collinearity test for all-cause death between the pre-PCI TIMI 0/1 and pre-PCI TIMI 2/3 groups

| | Variance Inflation Factors | Tolerance | Condition Index |
|---|---|---|---|
| Male | 1.247 | 0.802 | 1.000 |
| Age | 2.504 | 0.399 | 3.243 |
| LVEF | 1.315 | 0.761 | 3.334 |
| BMI | 1.211 | 0.826 | 3.421 |
| SBP | 3.181 | 0.314 | 3.928 |
| DBP | 2.933 | 0.341 | 4.329 |
| Cardiogenic shock | 1.291 | 0.774 | 4.371 |
| CPR on admission | 1.520 | 0.658 | 3.788 |
| SDT | 1.035 | 0.966 | 3.932 |
| DBT | 1.084 | 0.923 | 3.991 |
| Atypical chest pain | 1.198 | 0.835 | 4.202 |
| Dyspnea | 1.246 | 0.802 | 4.258 |
| Q-wave | 1.038 | 0.963 | 5.015 |
| Killip class II/III | 1.579 | 0.633 | 5.128 |
| Hypertension | 1.204 | 0.830 | 5.457 |
| Diabetes mellitus | 1.146 | 0.872 | 5.311 |
| Dyslipidemia | 1.044 | 0.958 | 5.627 |
| Peak CK-MB | 1.195 | 0.837 | 5.705 |
| Peak troponin-I | 1.225 | 0.817 | 6.057 |
| Total cholesterol | 4.335 | 0.231 | 6.094 |
| LDL-cholesterol | 4.250 | 0.235 | 6.322 |
| GRACE risk score | 3.684 | 0.271 | 6.831 |
| Ticagrelor | 1.061 | 0.943 | 7.279 |
| Left main (IRA) | 3.043 | 0.329 | 8.551 |
| LAD (IRA) | 3.641 | 0.275 | 9.253 |
| LCx (IRA) | 4.560 | 0.219 | 9.368 |
| RCA (IRA) | 2.878 | 0.347 | 10.219 |
| Left main (treated vessel) | 2.241 | 0.425 | 11.690 |
| LAD (treated vessel) | 2.321 | 0.431 | 12.427 |
| LCx (treated vessel) | 3.122 | 0.446 | 13.224 |
| RCA (treated vessel) | 1.085 | 0.320 | 16.255 |
| ACC/AHA type B2/C lesions | 1.108 | 0.922 | 22.012 |
| Transradial approach | 1.085 | 0.902 | 22.715 |
| GP IIb/IIIa inhibitor, n (%) | 1.514 | 0.934 | 24.089 |
| IVUS/OCT | 1.108 | 0.907 | 25.542 |
| FFR | 1.104 | 0.987 | 28.833 |
| 1$^{st}$-generation DES | 2.083 | 0.480 | 36.509 |
| 2$^{nd}$-generation DES | 2.165 | 0.462 | 42.186 |
| Stent diameter | 1.221 | 0.819 | 45.193 |
| Stent length | 2.602 | 0.384 | 55.564 |
| Number of stents | 2.415 | 0.414 | 90.710 |

Pre-PCI TIMI, pre-percutaneous coronary intervention thrombolysis in myocardial infarction flow grade; LVEF, left ventricular ejection fraction; BMI, body mass index; SBP, systolic blood pressure; DBP, diastolic blood pressure; CPR, cardiopulmonary resuscitation; SDT, symptom-

to-door time; DBT, door-to-balloon time; CK-MB, creatine kinase myocardial band; LDL, low-density lipoprotein; GRACE, Global Registry of Acute Coronary Events; IRA, infarct-related artery; LAD, left anterior descending coronary artery; LCx, left circumflex coronary artery; RCA, right coronary artery; ACC/AHA, American College of Cardiology/American Heart Association; IVUS, intravascular ultrasound; OCT, optical coherence tomography; FFR, fractional flow reserve; DES, drug-eluting stent.

**Table S4.** Baseline characteristics between the pre-PCI TIMI 0/1 and pre-PCI TIMI 2/3 groups in patients with SBT <48 h or ≥48 h

| Variables | SBT <48 h (n = 3293) | | | SBT ≥48 h (n = 1617) | | |
|---|---|---|---|---|---|---|
| | Pre-TIMI 0/1 (*n* = 1328, group A) | Pre-TIMI 2/3 (*n* = 1965, group C) | *p* value | Pre-TIMI 0/1 (*n* = 558, group B) | Pre-TIMI 2/3 (*n* = 1059, group D) | *p* value |
| Male, *n* (%) | 996 (75.5) | 1436 (73.1) | 0.225 | 395 (70.8) | 723 (68.3) | 0.308 |
| Age, years | 62.5 ± 12.2 | 64.4 ± 11.8 | <0.001 | 66.3 ± 12.4 | 66.8 ± 12.0 | 0.430 |
| LVEF, % | 53.4 ± 9.8 | 55.2 ± 10.9 | <0.001 | 50.3 ± 9.4 | 54.1 ± 11.6 | <0.001 |
| BMI, kg/m$^2$ | 24.2 ± 3.4 | 24.0 ± 3.3 | 0.023 | 23.9 ± 3.3 | 24.1 ± 3.5 | 0.282 |
| SBP, mmHg | 133.7 ± 26.7 | 136.0 ± 26.5 | 0.015 | 133.0 ± 28.0 | 137.1 ± 24.6 | 0.003 |
| DBP, mmHg | 81.0 ± 16.4 | 81.8 ± 15.4 | 0.185 | 79.7 ± 16.0 | 81.3 ± 14.4 | 0.039 |
| Cardiogenic shock, *n* (%) | 71 (5.3) | 96 (4.9) | 0.571 | 19 (3.4) | 34 (3.2) | 0.883 |
| CPR on admission, *n* (%) | 39 (2.9) | 64 (3.3) | 0.683 | 21 (3.8) | 35 (3.3) | 0.668 |
| SDT, hours | 4.7 (2.0-11.3) | 4.0 (1.8-9.3) | 0.004 | 65.0 (21.5-114.2) | 48.0 (9.1-101.0) | 0.165 |
| DBT, hours | 6.2 (2.8-16.0) | 10.8 (3.6-19.9) | <0.001 | 24.6 (6.0-56.5) | 39.1 (16.7-63.2) | 0.133 |
| Atypical chest pain, *n* (%) | 178 (13.4) | 275 (14.0) | 0.643 | 137 (24.6) | 224 (21.2) | 0.132 |
| Dyspnea, *n* (%) | 297 (22.4) | 413 (21.0) | 0.365 | 202 (36.2) | 323 (30.5) | 0.022 |
| EKG on admission | | | | | | |
| Q-wave, *n* (%) | 133 (10.0) | 111 (5.6) | <0.001 | 63 (11.3) | 91 (8.6) | 0.090 |
| ST-segment depression, *n* (%) | 308 (23.2) | 465 (23.7) | 0.769 | 112 (20.1) | 225 (21.2) | 0.607 |
| T-wave inversion, *n* (%) | 279 (21.0) | 437 (22.2) | 0.413 | 144 (25.8) | 285 (26.9) | 0.678 |
| Atrial fibrillation, *n* (%) | 48 (3.6) | 86 (4.4) | 0.323 | 25 (4.5) | 49 (4.6) | 0.893 |
| Killip class 1I/III, *n* (%) | 225 (16.9) | 249 (12.7) | 0.001 | 143 (25.6) | 182 (17.2) | <0.001 |
| First medical contact | | | | | | |
| EMS, *n* (%) | 167 (12.6) | 230 (11.7) | 0.478 | 31 (5.6) | 69 (6.5) | 0.515 |
| Non-PCI center, *n* (%) | 698 (52.6) | 1047 (53.3) | 0.696 | 310 (55.6) | 574 (54.2) | 0.636 |
| PCI center, *n* (%) | 463 (34.9) | 688 (35.0) | 0.941 | 217 (38.9) | 416 (39.3) | 0.915 |
| Hypertension, *n* (%) | 638 (48.0) | 1077 (54.8) | <0.001 | 293 (52.5) | 617 (58.3) | 0.027 |
| Diabetes mellitus, *n* (%) | 334 (25.2) | 631 (32.1) | <0.001 | 176 (31.5) | 359 (33.9) | 0.345 |
| Dyslipidemia, *n* (%) | 139 (10.5) | 258 (13.1) | 0.022 | 59 (10.6) | 129 (12.2) | 0.370 |

| | | | | | | |
|---|---|---|---|---|---|---|
| Previous MI, *n* (%) | 97 (7.3) | 132 (6.7) | 0.530 | 33 (5.9) | 81 (7.6) | 0.220 |
| Previous PCI, *n* (%) | 132 (9.9) | 196 (10.0) | 0.974 | 48 (8.6) | 108 (10.2) | 0.330 |
| Previous CABG, *n* (%) | 6 (0.5) | 19 (1.0) | 0.095 | 5 (0.9) | 10 (0.9) | 0.923 |
| Previous HF, *n* (%) | 14 (1.1) | 36 (1.8) | 0.082 | 7 (1.3) | 23 (2.2) | 0.246 |
| Previous stroke, *n* (%) | 68 (5.1) | 119 (6.1) | 0.283 | 37 (6.6) | 81 (7.6) | 0.483 |
| Current smokers, *n* (%) | 530 (39.9) | 732 (37.3) | 0.125 | 175 (31.5) | 332 (31.4) | 0.955 |
| Peak CK-MB, mg/dL | 61.2 (11.5-153.9) | 19.8 (6.5-65.5) | <0.001 | 15.0 (5.0-51.8) | 10.8 (4.5-32.2) | 0.079 |
| Peak troponin-I, ng/mL | 19.1 (4.8-42.7) | 5.4 (1.2-20.7) | <0.001 | 7.1 (2.2-20.8) | 3.0 (0.8-10.6) | <0.001 |
| Serum creatinine (mg/L) | 1.10 ± 1.28 | 1.14 ± 1.37 | 0.390 | 1.23 ± 1.50 | 1.18 ± 1.29 | 0.455 |
| Total cholesterol, mg/dL | 186.6 ± 46.8 | 176.1 ± 45.1 | <0.001 | 179.5 ± 45.5 | 173.5 ± 44.9 | 0.013 |
| Triglyceride, mg/L | 136.1 ± 125.2 | 133.2 ± 137.4 | 0.552 | 128.2 ± 116.0 | 128.8 ± 90.9 | 0.922 |
| HDL cholesterol, mg/L | 42.9 ± 11.5 | 42.9 ± 11.7 | 0.975 | 42.0 ± 11.8 | 42.3 ± 11.9 | 0.601 |
| LDL cholesterol, mg/L | 118.4 ± 39.8 | 111.2 ± 38.5 | <0.001 | 112.5 ± 38.1 | 109.0 ± 39.2 | 0.108 |
| GRACE risk score | 128.7 ± 42.4 | 130.9 ± 41.2 | 0.147 | 139.2 ± 43.3 | 134.5 ± 38.8 | 0.031 |
| Discharge medications, *n* (%) | | | | | | |
| Aspirin, *n* (%) | 1316 (99.1) | 1942 (98.8) | 0.494 | 552 (98.9) | 1049 (99.1) | 0.796 |
| Clopidogrel, *n* (%) | 951 (71.6) | 1365 (69.5) | 0.199 | 428 (76.7) | 773 (73.0) | 0.107 |
| Ticagrelor, *n* (%) | 239 (18.0) | 418 (21.3) | 0.021 | 82 (14.7) | 207 (19.5) | 0.017 |
| Prasugrel, *n* (%) | 138 (10.4) | 182 (9.3) | 0.308 | 48 (8.6) | 79 (7.5) | 0.437 |
| BBs, *n* (%) | 1132 (85.2) | 1666 (84.8) | 0.728 | 456 (81.7) | 882 (83.3) | 0.447 |
| ACEI or ARBs, *n* (%) | 1097 (82.6) | 1648 (83.9) | 0.341 | 446 (79.9) | 879 (83.0) | 0.135 |
| Statin, *n* (%) | 1251 (94.2) | 1868 (95.1) | 0.302 | 515 (92.3) | 993 (93.8) | 0.297 |
| Anticoagulant, *n* (%) | 38 (2.9) | 43 (2.2) | 0.251 | 22 (3.9) | 33 (3.1) | 0.389 |
| Infarct-related artery | | | | | | |
| Left main, *n* (%) | 12 (0.9) | 79 (4.0) | <0.001 | 7 (1.3) | 50 (4.7) | <0.001 |
| LAD, *n* (%) | 410 (30.9) | 966 (49.2) | <0.001 | 195 (34.9) | 505 (47.7) | <0.001 |
| LCx, *n* (%) | 446 (33.6) | 450 (22.9) | <0.001 | 128 (22.9) | 235 (22.2) | 0.754 |
| RCA, *n* (%) | 460 (34.6) | 470 (23.9) | <0.001 | 215 (38.5) | 269 (25.4) | <0.001 |
| Treated vessel | | | | | | |
| Left main, *n* (%) | 28 (2.1) | 106 (5.4) | <0.001 | 20 (3.6) | 69 (6.5) | 0.016 |

| | | | | | | |
|---|---|---|---|---|---|---|
| LAD, *n* (%) | 605 (45.6) | 1227 (62.4) | <0.001 | 299 (53.6) | 668 (63.1) | <0.001 |
| LCx, *n* (%) | 597 (45.0) | 702 (35.7) | <0.001 | 209 (37.5) | 380 (35.9) | 0.550 |
| RCA, *n* (%) | 565 (42.5) | 651 (33.1) | <0.001 | 257 (46.1) | 372 (35.1) | <0.001 |
| ACC/AHA type B2/C lesions, *n* (%) | 1148 (86.4) | 1656 (84.3) | 0.090 | 493 (88.4) | 863 (81.5) | 0.001 |
| Transradial approach, *n* (%) | 567 (42.7) | 1111 (56.5) | <0.001 | 270 (48.4) | 597 (56.4) | 0.002 |
| GP IIb/IIIa inhibitor, *n* (%) | 193 (14.5) | 113 (5.8) | <0.001 | 74 (13.3) | 53 (5.0) | <0.001 |
| IVUS/OCT, *n* (%) | 236 (17.8) | 553 (28.1) | <0.001 | 135 (24.2) | 317 (29.9) | 0.014 |
| FFR, *n* (%) | 15 (1.1) | 44 (2.2) | 0.022 | 10 (1.8) | 36 (3.4) | 0.082 |
| Stents | | | | | | |
| Bare-metal stent, *n* (%) | 30 (2.3) | 66 (3.4) | 0.073 | 18 (3.2) | 42 (4.0) | 0.492 |
| $1^{st}$-generation DES, *n* (%) | 43 (3.2) | 79 (4.0) | 0.260 | 13 (2.3) | 52 (4.9) | 0.011 |
| $2^{nd}$-generation DES, *n* (%) | 1255 (94.5) | 1820 (92.6) | 0.038 | 527 (94.4) | 965 (91.1) | 0.019 |
| Stent diameter (mm) | 3.04 ± 0.42 | 3.12 ± 0.44 | <0.001 | 3.02 ± 0.40 | 3.09 ± 0.44 | 0.001 |
| Stent length (mm) | 30.4 ± 13.8 | 28.0 ± 13.1 | <0.001 | 32.7 ± 16.2 | 29.3 ± 14.3 | <0.001 |
| Number of stents | 1.21 ± 0.47 | 1.18 ± 0.42 | 0.023 | 1.26 ± 0.51 | 1.22 ± 0.48 | 0.155 |

Values are means ± standard deviation or median (interquartile range) or numbers and percentages. The *p* values for continuous data were obtained from the unpaired t-test. The *p* values for categorical data were obtained from the chi-square or Fisher's exact test. Pre-PCI TIMI, pre-percutaneous coronary intervention thrombolysis in myocardial infarction flow grade; SBT, symptom-to-balloon time; LVEF, left ventricular ejection fraction; BMI, body mass index; SBP, systolic blood pressure; DBP, diastolic blood pressure; CPR, cardiopulmonary resuscitation; SDT, symptom-to-door time; DBT, door-to-balloon time; EKG, electrocardiogram; EMS, emergency medical service; MI, myocardial infarction; CABG, coronary artery bypass graft; HF, heart failure; CK-MB, creatine kinase myocardial band; HDL, high-density lipoprotein; LDL, low-density lipoprotein; GRACE, Global Registry of Acute Coronary Events; BBs, beta-blockers; ACEIs, angiotensin converting enzyme inhibitors; ARBs, angiotensin receptor blockers; LAD, left anterior descending coronary artery; LCx, left circumflex coronary artery; RCA, right coronary artery; ACC/AHA, American College of Cardiology/American Heart Association; GP, glycoprotein; IVUS/OCT, intravascular ultrasound/optical coherence tomography; FFR, fractional flow reserve; DES, drug-eluting stent.
